# Cervical Cancer Cell Line Secretome Highlights the Roles of Transforming Growth Factor-Beta-Induced Protein ig-h3, Peroxiredoxin-2, and NRF2 on Cervical Carcinogenesis

**DOI:** 10.1155/2017/4180703

**Published:** 2017-02-02

**Authors:** Georgia Kontostathi, Jerome Zoidakis, Manousos Makridakis, Vasiliki Lygirou, George Mermelekas, Theofilos Papadopoulos, Konstantinos Vougas, Alexios Vlamis-Gardikas, Peter Drakakis, Dimitrios Loutradis, Antonia Vlahou, Nicholas P. Anagnou, Kalliopi I. Pappa

**Affiliations:** ^1^Biotechnology Division, Biomedical Research Foundation, Academy of Athens (BRFAA), Athens, Greece; ^2^Laboratory of Biology, University of Athens School of Medicine, Athens, Greece; ^3^Institut National de la Santé et de la Recherche Médicale (INSERM), U1048, Institute of Cardiovascular and Metabolic Disease, Toulouse, France; ^4^Université Toulouse III Paul-Sabatier, Toulouse, France; ^5^Department of Chemistry, Division of Organic Chemistry, Biochemistry and Natural Products, University of Patras, 26504 Rion, Greece; ^6^First Department of Obstetrics and Gynecology, University of Athens School of Medicine, Alexandra Hospital, Athens, Greece; ^7^Laboratory of Cell and Gene Therapy, Biomedical Research Foundation, Academy of Athens, Athens, Greece

## Abstract

Cancer cells acquire unique secretome compositions that contribute to tumor development and metastasis. The aim of our study was to elucidate the biological processes involved in cervical cancer, by performing a proteomic analysis of the secretome from the following informative cervical cell lines: SiHa (HPV16+), HeLa (HPV18+), C33A (HPV−), and HCK1T (normal). Proteins were analyzed by 2D gel electrophoresis coupled to MALDI-TOF-MS. Enrichment of secreted proteins with characteristic profiles for each cell line was followed by the identification of differentially expressed proteins. Particularly, transforming growth factor-beta-induced protein ig-h3 (Beta ig-h3) and peroxiredoxin-2 (PRDX2) overexpression in the secretome of cancer cell lines was detected and confirmed by Western blot. Bioinformatics analysis identified the transcription factor NRF2 as a regulator of differentially expressed proteins in the cervical cancer secretome. NRF2 levels were measured by both Western blot and Multiple Reaction Monitoring (MRM) in the total cell extract of the four cell lines. NRF2 was upregulated in SiHa and C33A compared to HCK1T. In conclusion, the secreted proteins identified in cervical cancer cell lines indicate that aberrant NRF2-mediated oxidative stress response (OSR) is a prominent feature of cervical carcinogenesis.

## 1. Introduction

Cervical cancer belongs to a group of gynecological cancers, including vulvar and endometrial cancer that share common features, such as differentially expressed proteins, pathways, and transcription factors [1]. Cervical cancer is the fourth most common cancer in women across the world [2]. The majority of cervical cancer incidents are attributed to 13 high-risk oncogenic HPV types, represented mainly by HPV16 and HPV18. HPV infection of the cervical epithelium results in the eventual expression of E6 and E7 oncogenes, leading to sequential steps of tumor progression, corresponding to discrete histological lesions such as CIN1, CIN2, and CIN3 [3].

Infection of cervical epithelium with high-risk HPV types represents the initiating event towards cervical cancer. Proteomic studies are a valuable tool in order to explore the mechanisms involved in viral infection and protein dysfunction interplay that lead to cervical carcinogenesis [4]. Furthermore, proteomic approaches have been widely utilized for the discovery of novel putative biomarkers but also for understanding the mechanism of action of drugs in cervical cancer treatment [5].

Although a lot of clinical samples and cell lines have been used in proteomics studies [4, 5], novel proteomic approaches based on representative features of cancer cell phenotype must be employed. For example, a limitation of the current proteomics approaches is the lack of data on cervical cancer cell line secretomes [5]. The cell secretome represents the collection of the entire macromolecules secreted by a cell and constitutes a vital aspect of cell-cell communication. During carcinogenesis, cancer cells display secretomes with specific altered composition, reflecting the acquisition of the hallmarks of cancer with a potential contribution to the distinctive stages of cancer progression [6].

In the present study, we focused on the systematic evaluation of the secretome of representative cervical cancer cell lines in order to study the role of secreted proteins in cervical carcinogenesis. The secretome of a normal cervical keratinocytes cell line, HCK1T [7], was compared to the secretome of three informative cervical cancer cell lines [C33A (HPV negative), SiHa (HPV16+), and HeLa (HPV18+)]. The employment of such complementary cell lines offers a detailed and reliable comparison, since the effects of the most common HPV types that are responsible for cervical cancer (types 16 and 18) were assessed versus HPV negative and normal cervical cells. Specifically, the use of the C33A cancer cell line which is HPV negative was employed in order to offer a comprehensive coverage of the cervical cancer cell phenotype in the absence of HPV. Finally, HCK1T represents an appropriate control, as it originates from normal human cervical keratinocytes. To our knowledge, this is the first time that such a reference cell line has been incorporated in cervical cancer proteomic studies, since only cell lines deriving from human foreskin keratinocytes have been used as normal control previously [8]. The two-dimensional gel electrophoresis (2DE) analysis revealed proteomic changes among the cell lines, including classically and nonclassically secreted proteins, such as the transforming growth factor-beta-induced protein ig-h3 (Beta ig-h3) and peroxiredoxin-2 (PRDX2). A detailed bioinformatics analysis was also performed in order to reveal the altered pathways and upstream transcription factors that may be inducing such proteomic changes, which eventually highlighted the potential involvement of NRF2.

## 2. Materials and Methods

### 2.1. Cell Culture and Sample Preparation for Proteomics Analysis

SiHa, HeLa, and C33A cells were purchased from ATCC and cultured in DMEM, supplemented with 10% FBS, 1% P/S (supplied by Gibco-Invitrogen) at 37°C, and 5% CO_2_, as previously described [9]. ΗCK1T cells were a kind gift of Dr. Tohru Kiyono [7] and were cultured as proposed [10] in Defined Keratinocyte Serum-Free Medium (Gibco-Invitrogen), supplemented with 5 ng/mL EGF (Epidermal Growth Factor; Gibco-Invitrogen) and 50 *μ*g/mL of BPE (Bovine Pituitary Extract; Gibco-Invitrogen). The secretome or conditioned medium (CM) as well as the total cell extract was collected as previously described by us [11]. Briefly, the secretome of the cell lines was collected as follows: the medium in which the cell lines were propagated (DMEM for cancer cell lines and Defined Keratinocyte Serum-Free Medium for HCK1T) was removed when the cells reached a concentration of 10^6^ cells per mL (80–90% confluency). The cell layer was washed 3 times with 1x PBS (Gibco-Invitrogen) and once with DMEM-Serum and Phenol Red Free Medium (SFM) (Gibco-Invitrogen). SFM was then added to the cells for an incubation period of 24 h after which the SFM was collected.

### 2.2. Two-Dimensional Gel Electrophoresis (2DE)

CM was analyzed by 2DE according to Chevallet et al. [12]. Proteins (60 *μ*g) were resolved on 7 cm nonlinear IPG strips, pH range 3–10 (Bio-Rad), using the in-gel rehydration method. This was followed by a reduction (dithioerythritol) and alkylation (iodoacetamide) of IPG strips, while the second dimensional analysis was performed on 11% SDS-PAGE. Staining of 2DE gels was performed with Coomassie Colloidal Blue. Four biological replicates were analyzed for each cell line.

### 2.3. Spot Quantification

Spot quantification was performed as previously described [11]. Gels were scanned at a GS-800 imaging densitometer (Bio-Rad) in transmission mode and the images were analyzed using the PD Quest 8 software package (Bio-Rad). Normalization of the individual protein spot quantity was performed according to the total density in gel image and was expressed as ppm. Comparison of the expression level of the various proteins spots was performed employing the Mann–Whitney statistical test. Due to the relatively low statistical power of the experiment (*n* = 4 per cell line), protein spots with fold change >2 were considered as differentially expressed and included in further analysis. However, in all cases Mann–Whitney test was also applied and results with a *p* value of <0.05 were considered as statistically significant.

### 2.4. MALDI-TOF-MS (Matrix Assisted Laser Desorption Ionization-Time of Flight-Mass Spectrometry)

MALDI-TOF-MS was performed as previously described [11]. In brief, peptide masses were determined by MALDI-TOF-MS (Ultraflex TOF/TOF, Bruker Daltonics), peak list was created with Flexanalysis v2.2 software (Bruker Daltonics), smoothing was applied with Savitzky-Golay algorithm (width 0.2* m/z*, cycle number 1), and a signal/noise threshold ratio of 2.5 was allowed. For peptide matching (Mascot Server 2; Matrix Science), the following settings were used: monoisotopic mass, one miscleavage site allowed, carbamidomethylation of cysteine as fixed, and oxidation of methionine as variable modifications. Stringent criteria were used for protein identification with a maximum allowed mass error of 25 ppm and a minimum of 4 matching peptides. Notably, a large percentage of the proteins were identified based on six matches. The probability of a false identity was usually lower than 10^−5^. Analysis of the data was performed using a sequence-scrambled version of Swiss-Prot, generated by the decoy generating-script available at Matrix Science, using the settings described above, provided there are no identifications.

### 2.5. Western Blot Analysis

A protein amount of 10 *μ*g of secretome from SiHa, HeLa, C33A, and HCK1T cell lines or 20 *μ*g of total cell extract from the above cell lines was separated by 4–12% precast Bis-Tris gels Novex NU-PAGE (Gibco-Invitrogen), under reducing conditions and electroblotted to Hybond-ECL nitrocellulose membrane (GE Healthcare Lifesciences). After blocking with 5% nonfat dried milk in TBST (20 mM Tris, pH 7.6, 137 mM NaCl, 0.1% Tween 20) for 2 h at room temperature, membranes were washed with TBST and incubated overnight at 4°C with the following primary antibodies, as applicable: mouse anti-human PRXII (monoclonal, Santa Cruz; dilution 1 : 250, catalogue number sc-59660, antigen full-length PRX II of human origin), mouse anti-human NRF2 (monoclonal, R&D Systems; dilution 1 : 500, catalogue number MAB3925, antigen* E. coli*-derived recombinant human Nrf2 Met17-Asn605), rabbit anti-human BIGH3 (polyclonal, ProteinTech; dilution 1 : 500, catalogue number 10188-1-AP, antigen Ag0241), and goat anti-human HSP 90*β* (polyclonal, Santa Cruz; dilution 1 : 200, catalogue number sc-1057, antigen C-terminus of HSP 90*β* of human origin). Membranes were then washed with TBST and incubated with goat anti-mouse (Santa Cruz; dilution 1 : 2000, catalogue number sc-2005, antigen mouse IgG) or donkey anti-rabbit (Amersham Biosciences; dilution 1 : 5000, catalogue number NA9340, antigen Rabbit IgG) or rabbit anti-goat (Sigma Aldrich; dilution 1 : 20000, catalogue number A5420, antigen Rabbit IgG) HRP-conjugated secondary antibody for 2 h at room temperature. For the analysis in the total cell extract, mouse anti-human tubulin (monoclonal, Sigma Aldrich; dilution 1 : 6000, catalogue number T6199, antigen chick brain tubulin) was used for the normalization as a loading control. In the case of secretome samples, equal loading was confirmed by Coomassie staining of replicate gels and Ponceau S staining of the nitrocellulose membrane. A final wash with TBST was performed and target protein was detected by the Enhanced Chemiluminescence (Perkin-Elmer LAS, Inc.) detection system. Films were scanned and images were analyzed using Quantity One software (Bio-Rad). Different secretome samples to the ones used for the 2DE analysis were employed. Four biological replicates were analyzed for each cell line. The *p* values were calculated using Student's* t*-test.

### 2.6. Multiple Reaction Monitoring (MRM) LC-MS/MS Sample Preparation

Peptides corresponding to 100 *μ*g of total cell extract protein were used for Multiple Reaction Monitoring (MRM) LC-MS/MS analysis as previously described [13]. Briefly, after reduction (10 mM dithioerythritol) and alkylation (50 mM iodoacetamide) the samples were digested with trypsin (1 : 100 w/w enzyme : protein ratio). The peptide mixture was desalted with Zip-tips (Thermo Scientific) and dried using a vacuum centrifuge. The dried peptides were solubilized in an appropriate volume of 0.1% formic acid (FA) to obtain a final concentration of 1 *μ*g/*μ*L and approximately 0.2 pmol/*μ*L of spiked in synthetic labeled peptide was also added (JPT Peptide Technologies). Different total cell extract samples to the ones used for the Western blot were employed for the validation of NRF2. Three biological replicates were analyzed for each cell line.

### 2.7. MRM LC-MS/MS Assay Design and Method Development

Liquid chromatography was performed using an Agilent 1200 series nanopump system (Agilent Technologies, Inc.), coupled with a C18 nanocolumn (150 mm × 75 *μ*m, particle size 3.5 *μ*m) purchased by Agilent. Peptide separation and elution was achieved with a 40 min 5–45% ACN/water 0.1% FA gradient at a flow rate of 300 nl/min. Four microliters of each sample was injected.

Tryptic peptides were analyzed on an AB/MDS Sciex 4000 QTRAP with a nanoelectrospray ionization source controlled by Analyst 1.5 software (Sciex). The mass spectrometer was operated in MRM mode, with the first (Q1) and third quadrupole (Q3) at 0.7 unit mass resolution. At least five transitions were recorded for each peptide. Optimum collision energies for each transition were automatically calculated by the Skyline software [14]. Detailed information about the acquisition method and the used parameters are provided in Table S1 in Supplementary Material available online at https://doi.org/10.1155/2017/4180703.

### 2.8. Peptide Selection for MRM LC-MS/MS

A proteotypic peptide for NRF2 was selected from the list of unique proteotypic peptides found in PeptideAtlas (http://www.peptideatlas.org) [15]. The final selection was based on the quality of the MS/MS spectrum of each peptide in the human spectral library (human_consensus_final_true_lib), downloaded from NIST (National Institute of Standards and Technology, http://www.nist.gov/), and on the score and number of observations in MS-based proteomics experiments as provided from PeptideAtlas (http://www.peptideatlas.org) [15]. One proteotypic peptide with 5 transitions was finally selected to be tested (Table S1). Data analysis was performed using Skyline software and all chromatograms were manually inspected to ensure the quality and accurate peak picking. Finally, the ratio of light/heavy (light: endogenous, heavy: synthetic) peptides was used for quantification (Table S2).

### 2.9. Classification of Secreted Proteins

SignalP 4.1 [16] and SecretomeP 2.0 [17] were used for the prediction of classical and nonclassical secretion of identified proteins. SignalP predicts the presence and location of signal peptide cleavage sites in amino acid sequences. The SecretomeP server provides predictions of nonclassical, that is, nonsignal peptide triggered protein secretion. The Panther Classification System (http://www.pantherdb.org/) was used for the classification of the identified proteins according to their molecular function.

### 2.10. Ingenuity Pathway Analysis (IPA)

The identified differentially expressed proteins were subjected to IPA analysis (IPA, QIAGEN, http://www.qiagen.com/ingenuity) in order to perform pathway analysis and upstream transcription factor analysis. A manually annotated database of protein interactions and metabolic reactions obtained from the scientific literature is included in the IPA. Entry names of differentially expressed proteins were converted to gene names after their entry in the Retrieve/ID mapping of the Uniprot database (http://www.uniprot.org/). The processed gene names were listed in MS Excel and imported into IPA to map the canonical pathways and generate biological networks. Data were submitted as fold change values (ratios) calculated against the control group (HCK1T). Hypothetical networks were built among the experimental proteins and the IPA database proteins. After running the core analysis, statistically significant (*p* ≤ 0.05, Fisher's exact test) canonical pathways were selected. The activation *z-*score algorithm was used by IPA in order to make predictions. The *z-*score algorithm is designed to produce either a prediction of activation or inhibition or no prediction and also to reduce the generation of significant predictions by random data.

## 3. Results

### 3.1. Analysis of Protein Differential Expression in the SiHa, HeLa, C33A, and HCK1T Secretome

**Figure 1:**
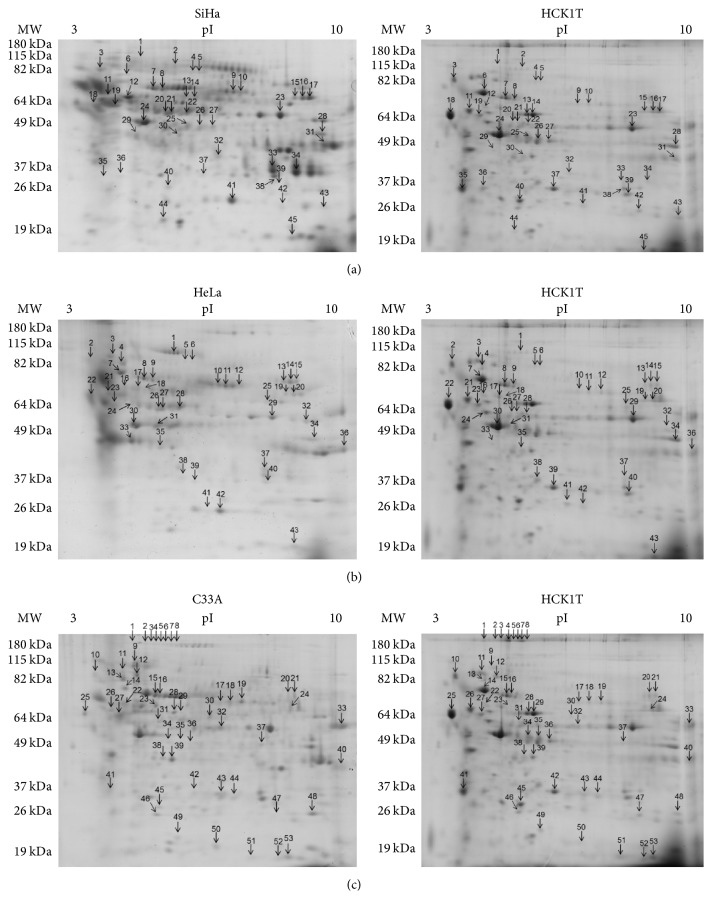
Representative 2D gel images showing the differentially expressed proteins in the secretome of cervical cancer cell lines versus the normal cancer cell line HCK1T. Representative 2D gels of each cancer cell line secretome (left) and HCK1T (right). Differentially expressed spots (over 2-fold), four gels per category corresponding to four biological replicates, are shown. (a) SiHa versus HCK1T comparison, (b) HeLa versus HCK1T comparison, (c) C33A versus HCK1T comparison. For the secretome analysis, 60 *μ*g of total protein was analyzed, using 7 cm nonlinear strips, pH range 3–10, and spot detection was performed by Coomassie Colloidal Blue staining. Protein identification was conducted by MALDI-TOF-MS.

**Figure 2:**
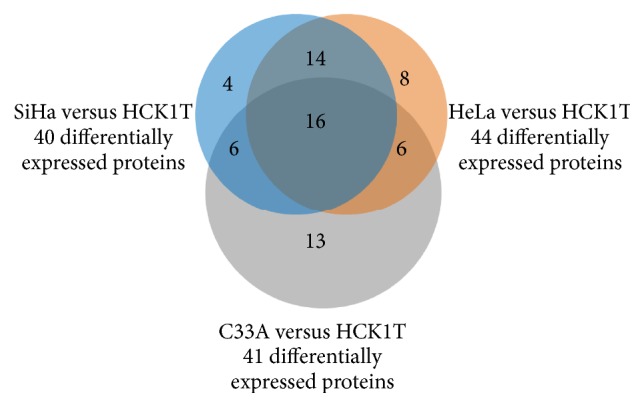
Venn diagram depicting the common proteins between the different comparisons (SiHa versus HCK1T, HeLa versus HCK1T, and C33A versus HCK1T).

Comparison of the expression levels of the proteins in the secretome (or conditioned media, CM) of the four cell lines was conducted, using a total of four gels per category, corresponding to different biological replicates. Each cancer cell line was compared to the normal cell line HCK1T. Representative gel images are shown in Figures 1(a), 1(b), and 1(c). In total, we identified 67 proteins, differentially expressed (fold change > 2) in cancer cell lines versus HCK1T (Table 1). We detected 45, 43, and 53 differentially expressed spots corresponding to 40, 44, and 41 proteins in SiHa versus HCK1T (Table S3), HeLa versus HCK1T (Table S4), and C33A versus HCK1T (Table S5), respectively. A Venn diagram (Figure 2) depicts the common differentially expressed proteins between the different comparisons (SiHa versus HCK1T, HeLa versus HCK1T, and C33A versus HCK1T). Sixteen proteins were found to be common in all comparisons and only 4, 8, and 13 proteins were unique in each individual comparison (SiHa versus HCK1T, HeLa versus HCK1T, and C33A versus HCK1T, resp.). Proteins used to create this Venn diagram are presented in Table S6. Only four proteins were upregulated in all three comparisons (SiHa versus HCK1T, HeLa versus HCK1T, and C33A versus HCK1T) and were differentially expressed at statistically significant levels (Mann–Whitney, *p* < 0.05). These were heat shock protein beta-1, nucleobindin-1, carboxypeptidase E, and calreticulin (Tables S3, S4, S5, and S6). Most of the secreted proteins were peptidases. Nucleobindin-1, carboxypeptidase E, and calreticulin are classically secreted (as described below), whereas heat shock protein beta-1 is not listed as classically secreted following bioinformatics analysis.

A total of 67 proteins were differentially expressed in the cancer cell lines versus HCK1T comparison based on the secretome analysis (Table 1). To confirm differences to total cell extract, a parallel analysis of the respective cell extracts was performed (Lygirou et al. in preparation). Following use of the SignalP software, 38.8% of the 67 proteins from the secretome analysis were predicted to be classically secreted, in comparison to 7.7% in the total cell extract.

The (in total) 67 differentially expressed proteins identified in the secretome of cancer cell lines compared to HCK1T were then categorized according to their molecular function, by the Panther Classification System (http://www.pantherdb.org/). The majority of proteins displayed catalytic activity (41.6%), while 32.4% displayed binding activity and 11.1% structural molecule activity (Figure S1). All the proteins and their molecular function are presented in Table S7. Furthermore, the molecular functions of the differentially expressed proteins in each individual cancer cell line (SiHa, HeLa, and C33A) compared to HCK1T were similar to the functions of the differentially expressed proteins from all three cancer cell lines versus HCK1T (Figure S2).

### 3.2. Validation of Quantitative Differences by Western Blot

**Figure 3:**
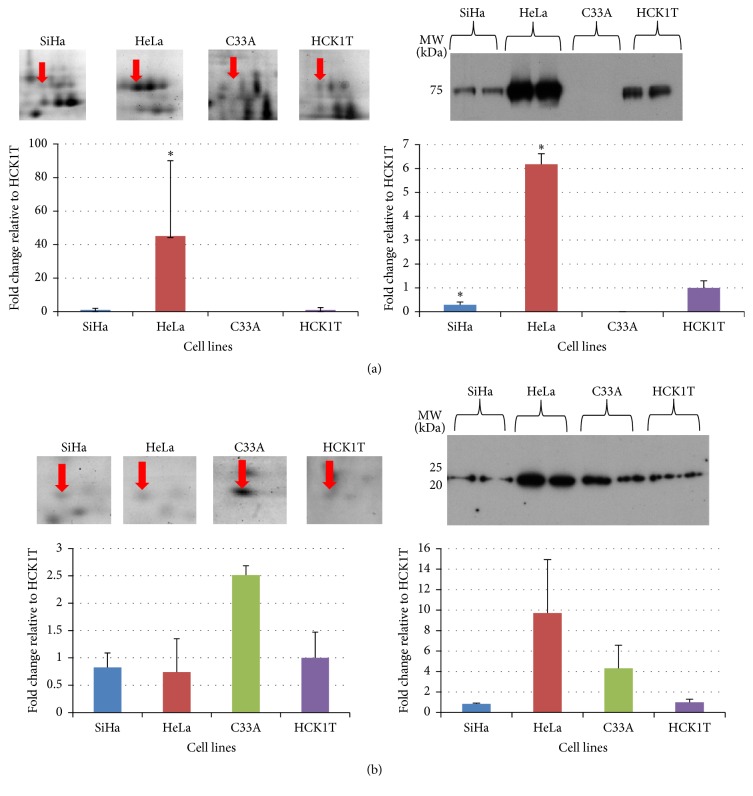
Validation of proteomics results by Western blot analysis. At the left side of each panel the proteomics results are presented and at the right side of the panel, the Western blot confirmation is shown. At the lower part of each panel, a graphical representation (fold change) is presented (mean ± SD, ^*∗*^*p* < 0.05, Mann–Whitney test or Student's* t*-test for 2D gels and Western blot, resp.). Red arrows indicate spots of interest in 2D gels and Western blot images are presented. Representative images of two biological replicates are shown for each cell line. (a) The upregulation of beta ig-h3 (transforming growth factor-beta-induced protein ig-h3) in HeLa cell lines compared to HCK1T observed in 2D gels (45.2 ± 44.9) was confirmed by Western blots for HeLa cells (6.2 ± 0.5). (b) The upregulation of PRDX2 (peroxiredoxin-2) in C33A cell line compared to HCK1T observed in 2D gels (2.5 ± 0.2) was confirmed by Western blot analysis (4.3 ± 2.3).

Among the proteins that were found to be upregulated in the cancer cell line secretome, transforming growth factor-beta-induced protein ig-h3 (beta ig-h3) and peroxiredoxin-2 (PRDX2) were the focal points of our study. These two proteins were selected for validation because they were differentially expressed in the secretome of several other cancer types when compared to controls [18, 19], as well as in cervical cancer tissues [20]. Beta ig-h3 is a classically secreted protein, whereas PRDX2 is a nonclassically secreted protein, according to SignalP and SecretomeP bioinformatics tools. Specifically, beta ig-h3 proteomics analysis showed an upregulation in HeLa (45.2-fold change, *p* < 0.05) whereas there was no difference in SiHa (1.0-fold change), when compared to HCK1T. Also the respective spot was not present in the C33A cell line (Figure 3(a), left panel). The upregulation of beta ig-h3 in HeLa versus HCK1T was further confirmed by Western blot analysis, as a band of approximately 75 kDa in the secretome (Figure 3(a), right panel). PRDX2 was upregulated in the C33A cell line when compared to HCK1T (2.5-fold change, *p* > 0.05) according to the proteomics analysis (Figure 3(b), left panel) while a protein band of 23 kDa was recognized by the specific antibody in the Western blot analysis, confirming the above upregulation (Figure 3(b), right panel). In contrast, in the SiHa and HeLa cell lines proteomics analysis, there was no difference when compared to HCK1T (0.8 and 0.7-fold change, resp.). In both cases, the observed molecular weight in the Western blot was in accordance with the 2D gels. Equal loading of the samples was confirmed by staining replicate SDS-PAGE gels with Coomassie Colloidal Blue (Figure S3). In order to ensure that peroxiredoxin-2 detected in the secretome was not the result of contamination due to cell lysis or cell death, the secretome from the cell lines was blotted with a tubulin antibody. Tubulin expression in the secretome was negligible in comparison to the corresponding total cell extract, thus confirming the origin of PRDX2 from the secretome. Furthermore, the percentage of necrotic cells in the secretome was <5% (Trypan Blue exclusion dye). Representative data are presented in Figure S4.

### 3.3. Ingenuity Pathway Analysis (IPA) and Validation of Bioinformatics Analysis by Western Blot

To further characterize the biological functions and the pathways involved in the regulation of the differentially expressed proteins, we employed the Ingenuity Pathway Analysis (IPA) software. Initially, we compared each cell line with one another and following these comparisons, we proceeded to the comparison of all the cancer cell lines versus the normal HCK1T, as the most representative approach. The differentially expressed proteins of all the cancer cell lines compared to HCK1T, as well as their corresponding entry names and their corresponding fold change, are presented in Table S8. This analysis was performed for the detection of putative direct or indirect interactions among proteins. Employing this approach, the most relevant associated canonical pathway was glycolysis (Table 2). In the top upstream transcription factors, p53, MYC, and MYCN, were highlighted as upstream regulators involved in the process of cancer (Table S9). NRF2 (NFE2L2) was revealed as an important upstream regulator which is responsible for the NRF2-mediated oxidative stress response (OSR) network function. The molecules that were included in important canonical pathways are also listed (Table 2).

**Figure 4:**
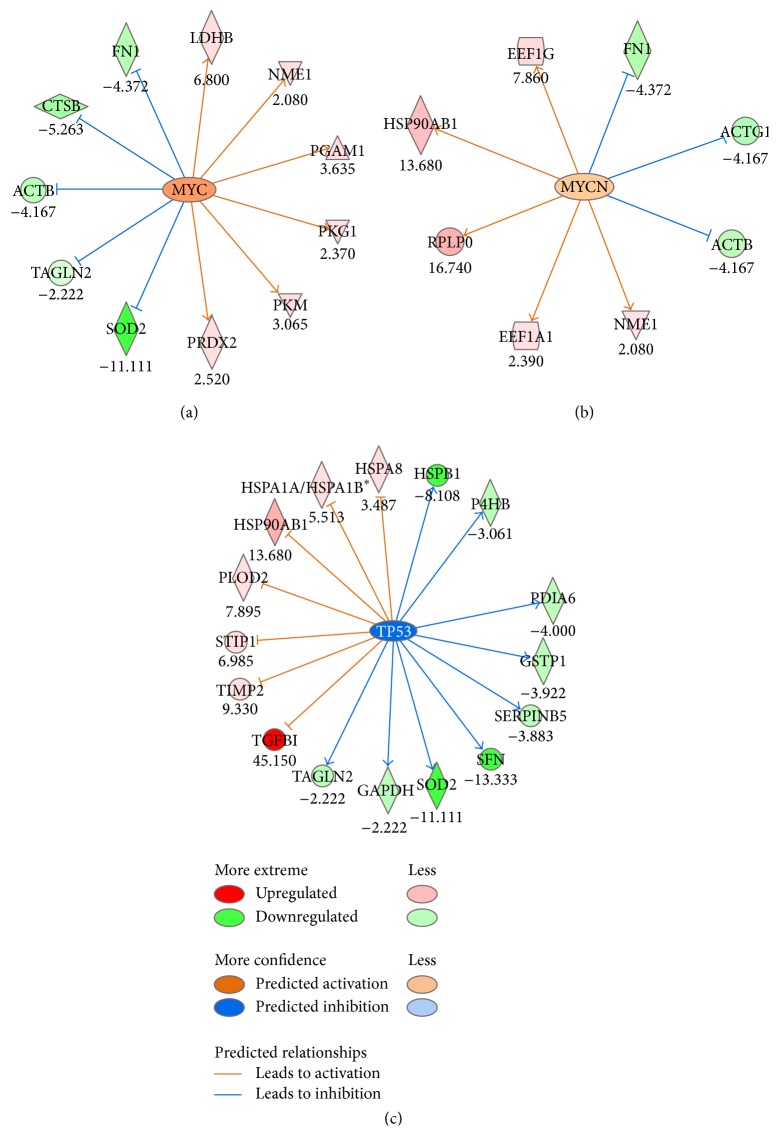
Representation of transcription factors (TFs) predicted to regulate genes based on differentially expressed proteins, according to IPA®. (a) The action of MYC and (b) MYCN shown as orange ellipse is predicted to be activated, whereas (c) T53, shown as blue ellipse, is predicted to be inhibited. Upregulated genes are colored in different shades of light pink to red, with darker color indicating higher degree of upregulation. Similarly, downregulated genes are shown in light to dark green, the latter indicating a higher degree of downregulation. The orange color of the arrows indicates activation, whereas the blue color indicates inhibition. ^*∗*^HSPA1A and HSPA1B genes correspond to the protein: Heat shock 70 kDa protein 1A/1B.

The top predicted transcription factors in our study, p53, MYC, and MYCN, are well-known upstream regulators involved in the process of carcinogenesis, since MYC and MYCN are oncogenes [21] and p53 is a tumor suppressor [22]. They affect several molecules among our differentially expressed proteins which are shown in Figure 4. The comparison of all cancer cell lines versus HCK1T revealed that MYCN as well as MYC were activated, and the majority of the downstream genes were upregulated. The output of IPA for transcription factors following the comparison of cancer cell lines versus HCK1T is presented in Table S9. Next, we examined a well-known suppressor of oncogenesis, the p53 protein, which is deregulated in cancer cell lines that contain HPV DNA, such as SiHa and HeLa. In the comparison of all cancer cell lines versus HCK1T, p53 was indeed predicted to be inhibited (*p* value 2.5 × 10^−15^, Fisher's exact test) (Table S9).

**Figure 5:**
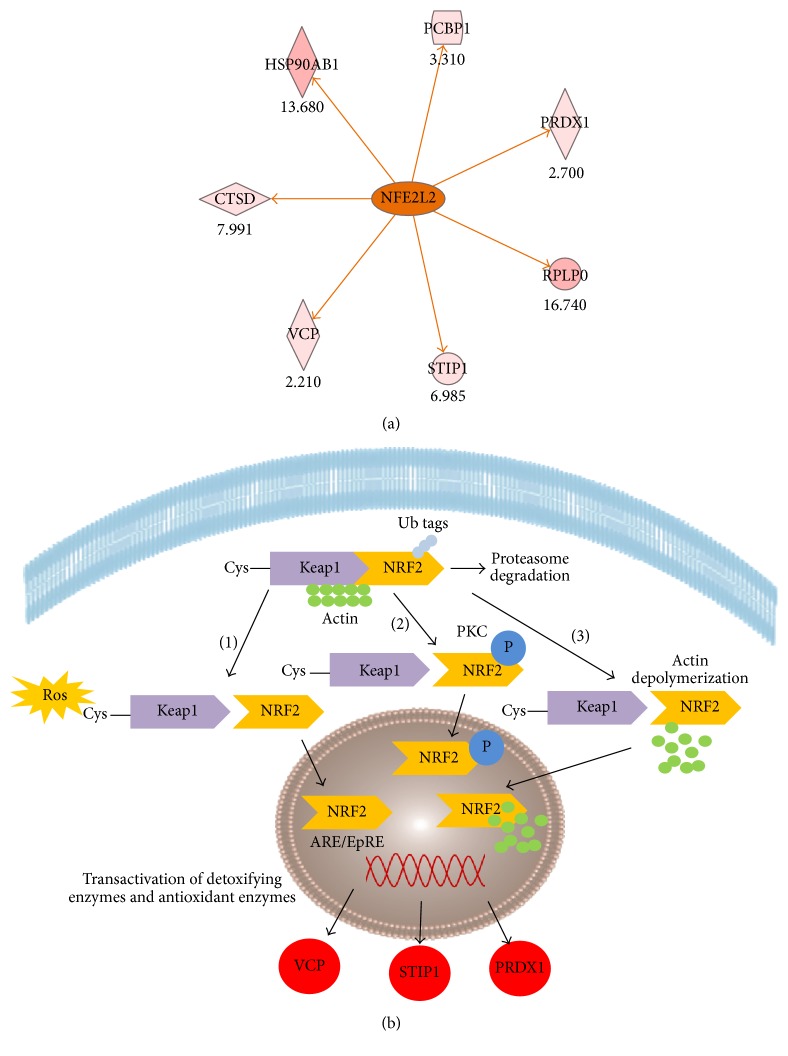
Schematic representation of NRF2 regulation and function, as predicted by IPA. (a) NRF2 or NFE2L2 (orange ellipse) action is predicted to be activated. All downstream genes are predicted to be upregulated and are colored in different shades of light pink to red, with darker color indicating higher degree of upregulation. The orange color indicates activation. (b) NRF2-mediated oxidative stress response is depicted. NRF2 binds to Keap1, following dependent (1), independent (2), or actin-dependent (3) mechanisms, described in detail in Discussion. NRF2 translocates to the nucleus, binds to antioxidant response elements (ARE/EpRE), and activates transcription of antioxidant and detoxifying enzymes, such as PRDX1 (peroxiredoxin-1), STIP1 (stress-induced-phosphoprotein-1), and VCP (transitional endoplasmic reticulum ATPase), which are upregulated (red color) according to the proteomics results.

The next important finding in the list of top transcription factors was the NRF2 transcription factor. NRF2-mediated oxidative stress response is included in the list of the important canonical pathways (Table 2). NRF2 is predicted to be activated, thus it can potentially upregulate several proteins. Such proteins as PRDX1 (peroxiredoxin-1), STIP1 (stress-induced-phosphoprotein 1), VCP (transitional endoplasmic reticulum ATPase), and CTSD (cathepsin D) which were differentially expressed in our analysis are included in the downstream targets that are upregulated by NRF2. Interestingly, the upregulation of most of them is also demonstrated in the pathway of NRF2-mediated oxidative stress response (Figures 5(a) and 5(b)).

**Figure 6:**
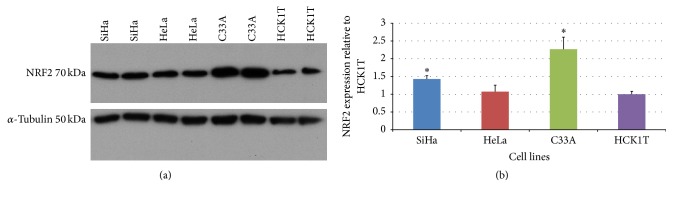
Confirmation of the IPA-predicted NRF2 activation in cancer cell lines by Western blot. Western blot analysis with NRF2-specific antibody in four different cell extracts per cell line (SiHa, HeLa, C33A, and HCK1T), corresponding to four biological replicates. Twenty *μ*g was loaded. (a) A protein band of 70 kDa corresponding to NRF2 is detected. Immunoblotting for *α*-tubulin (50 kDa) was applied to ensure the comparable loading of proteins in each lane. Fold expression of NRF2 was assessed relative to HCK1T. (b) The mean NRF2 values for SiHa, HeLa, and C33A were 1.4 ± 0.1 (*p* < 0.05), 1.1 ± 0.2 (*p* > 0.05), and 2.3 ± 0.3 (*p* < 0.05) compared to HCK1T, respectively. Representative images of two biological replicates are shown for each cell line. Graphical representation of densitometry analysis of the results (mean ± SD) is also shown (^*∗*^*p* < 0.05, Student's* t*-test).

In order to confirm the increased expression of NRF2 in the cervical cancer cell lines, we performed a Western blot analysis. Since NRF2 is not secreted, its expression validation was performed in total cell extracts. The actual upregulation of NRF2 in cancer cell lines versus HCK1T was confirmed as a band of approximately 70 kDa detected by the specific antibody (Figure 6(a)). The levels of NRF2 were higher in cancer cell lines, especially in C33A in comparison to HCK1T (fold change ~2.3, *p* < 0.05, Student's* t*-test), followed by a smaller upregulation in SiHa in comparison to HCK1T (fold change ~1.4, *p* < 0.05, Student's* t*-test) (Figure 6(b)). Equal loading of samples was confirmed by normalization with anti-tubulin antibody. Tubulin expression was confirmed as a band of 50 kDa.

To further document the effector role of NRF2, we validated by Western blot the differential expression of one protein that is predicted to be regulated by NRF2, according to the bioinformatics analysis illustrated in Figure 5(a). The expression of the selected protein [HSP90AB1 (heat shock protein HSP 90-beta)] was determined in C33A and HCK1T cells. HSP90AB1 was upregulated both in total cell extract analysis (fold change 2.3, *p* < 0.05, Student's* t*-test) (Figure S5 A) and in secretome analysis (fold change 4.8, *p* < 0.05, Student's* t*-test) (Figure S5 B) in C33A compared to HCK1T. Equal loading of samples was performed by normalization with anti-tubulin antibody in the case of total cell extract. Secretome equal loading was confirmed by staining with Coomassie Blue (Figure S3). The reported results strongly suggest that NRF2 could be the regulator of the above protein based on the agreement in its respective expression levels.

### 3.4. Validation of Bioinformatics Analysis by Multiple Reaction Monitoring (MRM)

**Figure 7:**
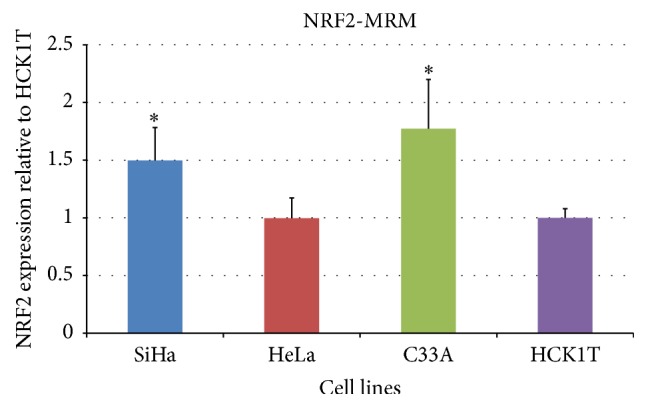
Confirmation of the IPA-predicted NRF2 activation in cancer cell lines by Multiple Reaction Monitoring (MRM). MRM analysis, performed in three different cell extracts from SiHa, HeLa, C33A, and HCK1T cells, corresponding to three biological replicates. One hundred *μ*g of protein was used for sample preparation. NRF2 fold expression (light to heavy peptide ratio) was assessed relative to HCK1T. The relative NRF2 expression for SiHa, HeLa, and C33A cell lines was 1.5 ± 0.3 (*p* < 0.05), 1.0 ± 0.2 (*p* > 0.05), and 1.8 ± 0.4 (*p* < 0.05) compared to HCK1T, respectively. Graphical representation of the results (mean ± SD) is shown (^*∗*^*p* < 0.05, Student's* t*-test).

In order to further increase the validity of the bioinformatics prediction regarding the activation of NRF2, we performed MRM in the total cell extracts. The higher upregulation of NRF2 in cancer cell lines versus HCK1T was mainly observed in the C33A cell line when compared to HCK1T (fold change ~1.8, *p* < 0.05, Student's* t*-test), followed by a lower upregulation in SiHa in comparison to HCK1T (fold change ~1.5, *p* < 0.05, Student's* t*-test) as shown in Figure 7. MRM specificity was ensured by the use of a reference heavy peptide (Table S1). Thus, MRM results were in full agreement with the Western blot results.

## 4. Discussion

Secreted proteins play a key role in cell signaling, communication, and migration. However, so far there are no studies exploring the potential role of secretome in cervical carcinogenesis. To our knowledge, our study represents the first report focusing on the secretome of both cervical cancer cell lines and normal cervical cells. Our protocol consisted of collecting secretome from confluent cultures of normal and cancer cell lines after an incubation period of 24 h in the presence of DMEM-Serum and Phenol Red Free Medium (SFM). The high percentage of secreted proteins identified (38.8%) verifies the efficiency of our protocol for the enrichment of secreted proteins.

An objective of this study was to explore the secretome-mediated processes that are involved in cervical carcinogenesis. Among a series of differentially expressed proteins, we detected two major proteins which were upregulated in cancer cell lines compared to the normal (Table 1). The first is the classically secreted transforming growth factor-beta-induced protein ig-h3 (beta ig-h3 or TGFBIp/*β*ig-h3), containing a signal peptide of 24 amino acids at the N-terminus. Beta ig-h3 is induced not only by TGF-*β* but also by other factors such as TNF-*α* and IL-1*β*. In the extracellular matrix, it is associated with collagen, fibronectin, laminin, and glycosaminoglycans and supports the adhesion of many cell types by recruiting integrins [23]. Beta ig-h3 was found to be upregulated in several cancer types such as colorectal cancer and renal cell carcinoma [18, 24]. In our study, beta ig-h3 was found to be upregulated in ΗeLa cell line secretome compared to HCK1T, which was further confirmed by Western blot analysis (Figure 3(a)).

In our analysis, we also focused on a nonclassically secreted protein such as peroxiredoxin-2 (PRDX2), as defined by SecretomeP. Peroxiredoxins (Prxs) are highly conserved antioxidant enzymes, involved in redox regulation of the cell, that fall into two major Prx subfamilies [25]. The role of cytoplasmic PRDX2 in cervical carcinogenesis was recently investigated. Immunohistochemical and immunoblot analysis of cervical cancer sections [20] revealed overexpression of peroxiredoxin-2 in the cancer samples when compared to controls. Furthermore, a study focused in breast cancer implied secretion of PRDX2 where tumor interstitial fluid (TIF) and normal interstitial fluid (NIF) from prospective cancer patients were compared, employing proteomic and immunohistochemistry analysis. PRDX2 was upregulated in TIF compared to NIF and was further validated by tissue microarray assays [19]. Our proteomic analysis documented that PRDX2 is indeed upregulated in the secretome of C33A cervical cell line versus HCK1T and this finding was confirmed by Western blot analysis (Figure 3(b)). The Western blot results show also a significant upregulation of PRDX2 in the HeLa secretome, but they are not in agreement with the proteomic analysis. This can be explained by the fact that, in the 2D gels, a single protein species of PRDX2 was identified and quantified, whereas the Western blot can probably detect multiple protein species that are upregulated in the HeLa secretome. In our study, PRDX2 is proposed as a nonclassically secreted protein in the context of cervical cancer (Figure S4), whereas previously it was reported as cytoplasmic [20].

IPA analysis pointed out NRF2 as a key transcription regulator and NRF2-mediated oxidative response as an important pathway in the cervical cancer cell lines. In order to verify the above bioinformatics prediction, we performed two independent analytical methods for validation, that is, Western blot and MRM analysis in the total cell extract of cell lines, where the potential activation of NRF2 actually takes place. The above methods yielded concurrent results. The expression of NRF2 was confirmed in the cervical cell lines, and it was upregulated in C33A and SiHa cancer cells compared to HCK1T (Figures 6 and 7).

In the pathway of NRF2-mediated oxidative stress response, several differentially expressed proteins are included, such as SOD2, PRDX1, ACTB, STIP1, VCP, ACTG1, and GSTP1 (Table 2). NRF2 is expected to upregulate the above proteins. However, only PRDX1, VCP, and STIP1 are upregulated according to the proteomic analysis (Table S8). This result confirms previous studies on the effect of NRF2 in the expression of these proteins [26–28]. In contrast, SOD2 and GSTP1 are downregulated in the cancer cell lines secretome, according to the proteomic analysis. In particular, PRDX1, VCP, and STIP are regulated by 6, 3, and 4 transcription factors, respectively, whereas SOD2 and GSTP1 are regulated by 28 and 12 transcription factors, respectively (Table S10). We can assume that NRF2 is the main transcription factor responsible for the upregulation of VCP, PRDX1, and STIP, whereas in the case of SOD2 and GSTP1, it is conceivable that there are additional transcription factors responsible for their downregulation. NRF2 has been proposed to act in cases as oncogene and in cases as tumor suppressor in cancer as it controls many biological functions. The most prominent role of NRF2 is the maintenance of redox homeostasis [29]. In our study, NRF2 acts as an oncogene, as it is upregulated in cervical cancer cell lines (SiHa and C33A) compared to the normal HCK1T. In line with our results, NRF2 was also shown to be upregulated in cervical cancer stem cells [30]. Moreover, knockdown of NRF2 has been performed in cervical cancer cell lines (CaSki, HeLa, and SiHa) [31–33]. In particular, Nrf2 stable knockdown by shRNA resulted in decreased expression of the NRF2/ARE-dependent detoxification and glutathione-related enzymes, like heme oxygenase-1 (HO-1) and NAD(P)H:quinone oxidoreductase 1 (NQO1) in CaSki cells [32]. The silencing of NRF2 resulted in increased cell apoptosis, decreased cell proliferation, migration, and invasion, which led to significant decrease of the malignant potential of SiHa cells [31].

Specifically, NRF2 silencing by siRNA inhibition reduced the expression of several antioxidant proteins, among them peroxiredoxin-1, in human scalp hair follicles (HFs), indicating that NRF2 protects human cells from oxidative damage [34]. In our study similarly, peroxiredoxin-1 was found upregulated in C33A versus HCK1T (NRF2 targets shown in Figure 5).

Along these lines HSP90AB1 is known to be regulated by NRF2 (NRF2 target shown in Figure 5(a)) which was found at increased levels in C33A versus HCK1T (in total cell extract lysates and in secretome, Figure S5), in agreement with the expression pattern of NRF2 (Figures 6 and 7). Furthermore, our experimental data show that peroxiredoxin-2 is upregulated in C33A versus HCK1T. A recent report proves that peroxiredoxin-2 expression is regulated by binding of NRF2 to the ARE elements of its promoter [35].

NRF2 is involved in the regulation of antioxidative genes and detoxifying enzymes, for the deactivation of reactive oxygen species or ROS [36], and interacts with the cytosolic Kelch-like ECH-associated protein 1 (Keap1) [37]. Under normal conditions, the above interaction leads NRF2 to proteasomal degradation through ubiquitination [38]. Under oxidative conditions, NRF2 is regulated through Keap1-dependent or Keap1-independent mechanisms. In Keap1-dependent mechanisms, cysteine residues in Keap1 are modified, resulting in conformational changes of the Keap1-NRF2 complex which inhibit Nrf2 ubiquitination and degradation [37]. In Keap1-independent mechanisms, NRF2 is phosphorylated by various kinases, for example, PKC (protein kinase C), disrupting its physical contact to Keap1 and leading to inhibition of Nrf2 ubiquitination and degradation [39, 40]. The translocation of NRF2 to the nucleus results in binding to antioxidant response elements (ARE/EpRE) and transcription activation of antioxidant and detoxifying enzymes [37]. In our IPA analysis, actin was found to be regulated by NRF2. Actin cytoskeleton has been reported to facilitate scaffolding of Keap1, as it binds to it, trapping NRF2 to the cytoplasm and thus preventing NRF2 translocation to the nucleus [41]. Moreover, activation of PI3-kinase signaling pathway rearranges actin microfilaments in response to oxidative stress, resulting in actin depolymerization, which leads to the formation and nuclear translocation of Nrf2-actin complexes in an actin-dependent mechanism [42], as shown in Figure 5(b).

Oxidative stress constitutes an important process in the context of cervical cancer as well. It has been suggested that ROS and high-risk HPVs can act synergistically in the onset and during the development of carcinogenesis [43]. Expression of HPV16 E7 oncoprotein in HaCaT human keratinocytes modifies the equilibrium between the oxidized and reduced forms of GSTP1, resulting in the inhibition of JNK phosphorylation and its ability to induce apoptosis [44]. In fact, GSTP1 was predicted to be regulated by NRF2 in our IPA analysis (Table 2 and Table S9), thus verifying the above connection of oxidative stress and cervical carcinogenesis.

## 5. Conclusions

Collectively, in the present study we performed a comprehensive comparison of the secreted proteins derived from three representative cervical cancer cell lines (SiHa, HeLa, and C33A) in regard to normal cervical keratinocytes (HCK1T) employing a combined proteomics and bioinformatics approach. This led to the identification of proteins associated with cervical cancer, such as beta ig-h3 and PRDX2, while bioinformatics analysis identified NRF2 as an important transcription regulator of secreted proteins; this in silico prediction was validated by the observed increase in NRF2 levels in cancer cells. Thus, NRF2 seems to play a pivotal role in cervical cancer and its precise function needs to be further investigated.

## Supplementary Material

Data regarding MRM (Multiple reaction monitoring) method development and quantification are presented in S1 Table and S2 Table respectively. The differentially expressed proteins for the following comparisons (SiHa vs HCK1T, HeLa vs HCK1T and C33A vs HCK1T) are presented in S3, S4 and S5 Tables, respectively. S6 Table shows the common differentially expressed proteins between the different comparisons in each cancer cell line vs HCK1T, as well as the unique proteins in each individual comparison. S7 Table depicts data analysis with Panther Classification System. S8-S10 Tables illustrate data from IPA bioinformatics analysis. S1 and S2 Figures illustrate the classification of differentially expressed proteins via Panther Classification System, based on molecular function. S3 Figure depicts equal loading of secretome samples utilized for Western blot analysis and S4 Figure illustrates the high quality of secretome samples, since the contamination of intracellular proteins (such as tubulin) as shown by Western blot analysis is minor. S5 Figure shows Western blots for HSP90AB in total cell extract and secretome.

## Figures and Tables

**Table 1:** Differentially expressed proteins in cancer versus HCK1T secretome. Sixty-seven proteins were found to be differentially expressed in cancer cell lines versus HCK1T in secretome analysis.

Entry name	Protein name	Cancer cell line of differential expression (compared to HCK1T)
1433S_HUMAN	14-3-3 protein sigma	SiHa, C33A
1A24_HUMAN	HLA class I histocompatibility antigen, A-24 alpha-chain	SiHa, HeLa
ACTB_HUMAN	Actin, cytoplasmic 1	SiHa, HeLa
ACTG_HUMAN	Actin, cytoplasmic 2	SiHa, HeLa
ALDOA_HUMAN	Fructose-bisphosphate aldolase A	SiHa, HeLa
C1R_HUMAN	Complement C1r subcomponent	SiHa, HeLa
CALR_HUMAN	Calreticulin	SiHa, HeLa, C33A
CATB_HUMAN	Cathepsin B	SiHa, C33A
CATD_HUMAN	Cathepsin D	SiHa, HeLa, C33A
CBPE_HUMAN	Carboxypeptidase E	SiHa, HeLa, C33A
ENOA_HUMAN	Alpha-enolase	SiHa, HeLa, C33A
FSTL4_HUMAN	Follistatin-related protein 4	SiHa, HeLa
G3P_HUMAN	Glyceraldehyde-3-phosphate dehydrogenase	SiHa
GANAB_HUMAN	Neutral alpha-glucosidase AB	SiHa, HeLa
GDIR1_HUMAN	Rho GDP-dissociation inhibitor 1	SiHa
GELS_HUMAN	Gelsolin	SiHa, HeLa
GLU2B_HUMAN	Glucosidase 2 subunit beta	SiHa, HeLa, C33A
GRP78_HUMAN	78 kDa glucose-regulated protein	SiHa, HeLa, C33A
GSTP1_HUMAN	Glutathione-S-transferase P	SiHa, C33A
HSP71_HUMAN	Heat shock 70 kDa protein 1A/1B	SiHa, HeLa, C33A
HSP7C_HUMAN	Heat shock cognate 71 kDa protein	SiHa, HeLa, C33A
HSPB1_HUMAN	Heat shock protein beta-1	SiHa, HeLa, C33A
K1C10_HUMAN	Keratin, type I cytoskeletal 10	SiHa, HeLa
K2C1_HUMAN	Keratin, type II cytoskeletal 1	SiHa, HeLa
KPYM_HUMAN	Pyruvate kinase PKM	SiHa, HeLa, C33A
NPC2_HUMAN	Epididymal secretory protein E1	SiHa
NUCB1_HUMAN	Nucleobindin-1	SiHa, HeLa, C33A
PCSK9_HUMAN	Proprotein convertase subtilisin/kexin type 9	SiHa, HeLa, C33A
PDIA1_HUMAN	Protein disulfide-isomerase	SiHa, HeLa, C33A
PDIA3_HUMAN	Protein disulfide-isomerase A3	SiHa, C33A
PGAM1_HUMAN	Phosphoglycerate mutase 1	SiHa, HeLa
PLOD2_HUMAN	Procollagen-lysine,2-oxoglutarate 5-dioxygenase 2	SiHa, HeLa
PPIA_HUMAN	Peptidyl-prolyl cis-trans isomerase A	SiHa, HeLa, C33A
SODM_HUMAN	Superoxide dismutase [Mn], mitochondrial	SiHa, C33A
SPB5_HUMAN	Serpin B5	SiHa, C33A
TAGL2_HUMAN	Transgelin-2	SiHa
TIMP1_HUMAN	Metalloproteinase inhibitor 1	SiHa, HeLa
TIMP2_HUMAN	Metalloproteinase inhibitor 2	SiHa, HeLa
TPIS_HUMAN	Triosephosphate isomerase	SiHa, HeLa, C33A
TPP1_HUMAN	Tripeptidyl-peptidase 1	SiHa, HeLa, C33A
ATPB_HUMAN	ATP synthase subunit beta, mitochondrial	HeLa
BGH3_HUMAN	Transforming growth factor-beta-induced protein ig-h3	HeLa
CATZ_HUMAN	Cathepsin Z	HeLa
DPP2_HUMAN	Dipeptidyl peptidase 2	HeLa
FSCN1_HUMAN	Fascin	HeLa
HS90B_HUMAN	Heat shock protein HSP 90-beta	HeLa, C33A
LAMC2_HUMAN	Laminin subunit gamma-2	HeLa, C33A
PARK7_HUMAN	Protein deglycase DJ-1	HeLa
PDIA6_HUMAN	Protein disulfide-isomerase A6	HeLa
PGK1_HUMAN	Phosphoglycerate kinase 1	HeLa
ROA1_HUMAN	Heterogeneous nuclear ribonucleoprotein A1	HeLa, C33A
STIP1_HUMAN	Stress-induced-phosphoprotein 1	HeLa, C33A
TCPQ_HUMAN	T-complex protein 1 subunit theta	HeLa, C33A
TKT_HUMAN	Transketolase	HeLa, C33A
COF1_HUMAN	Cofilin-1	C33A
EF1A1_HUMAN	Elongation factor 1-alpha 1	C33A
EF1G_HUMAN	Elongation factor 1-gamma	C33A
FINC_HUMAN	Fibronectin	C33A
HSP74_HUMAN	Heat shock 70 kDa protein 4	C33A
LDHB_HUMAN	L-lactate dehydrogenase B chain	C33A
NDKA_HUMAN	Nucleoside diphosphate kinase A	C33A
PCBP1_HUMAN	Poly(rC)-binding protein 1	C33A
PRDX1_HUMAN	Peroxiredoxin-1	C33A
PRDX2_HUMAN	Peroxiredoxin-2	C33A
PRDX6_HUMAN	Peroxiredoxin-6	C33A
RLA0_HUMAN	60S acidic ribosomal protein P0	C33A
TERA_HUMAN	Transitional endoplasmic reticulum ATPase	C33A

**Table 2:** Ingenuity pathway analysis-prediction of canonical pathways. Top canonical pathways and involved molecules as predicted by Ingenuity Pathway Analysis. Canonical pathways are classified according to *p* value (Fisher's exact test).

Canonical pathways	*p* value^a^	Ratio^b^	Molecules (gene name)
Glycolysis I	1.14 × 10^−12^	7/25 (28%)	PGK1, ENO1, TPI1, PGAM1, PKM, GAPDH, ALDOA
Unfolded protein response	3.89 × 10^−10^	7/54 (13%)	HSPA8, CALR, HSPA4, P4HB, HSPA1A/HSPA1B, VCP, HSPA5
Glyconeogenesis I	1.45 × 10^−8^	5/25 (20%)	PGK1, ENO1, PGAM1, GAPDH, ALDOA
Aldosterone signaling in epithelial cells	5.56 × 10^−7^	7/152 (4.6%)	HSPA8, HSPA4, HSP90AB1, PDIA3, HSPA1A/HSPA1B, HSPA5, HSPB1
NRF2-mediated oxidative stress response	1.73 × 10^−6^	7/178 (3.9%)	SOD2, PRDX1, ACTB, STIP1, VCP, ACTG1, GSTP1

^a^Fisher's exact test was used to calculate a *p *value for each protein of the data set identified in the biological function studied, indicating the probability that each biological function assigned to the data set is not assigned by chance. ^b^The Ratio of the canonical pathways is calculated based on the number of molecules from the input database divided by the total number of the molecules in the pathway that is predicted by IPA. Molecules participating in the important canonical pathways according to IPA analysis are listed by their gene names.
