# Supplementary material for: Cervical Cancer Cell Line Secretome Highlights the Roles of Transforming Growth Factor-Beta-Induced Protein ig-h3, Peroxiredoxin-2, and NRF2 on Cervical Carcinogenesis
Source: Biomed Res Int. 2017 Feb 2;2017:4180703. doi: 10.1155/2017/4180703 (PMC5316418; doi:10.1155/2017/4180703)
Supplement: Supplementary file 1 — Data regarding MRM (Multiple reaction monitoring) method development and quantification are presented in S1 Table and S2 Table respectively. The differentially expressed proteins for the following comparisons (SiHa vs HCK1T, HeLa vs HCK1T and C33A vs HCK1T) are presented in S3, S4 and S5 Tables, respectively. S6 Table shows the common differentially expressed proteins between the different comparisons in each cancer cell line vs HCK1T, as well as the unique proteins in each individual comparison. S7 Table depicts data analysis with Panther Classification System. S8-S10 Tables illustrate data from IPA bioinformatics analysis. S1 and S2 Figures illustrate the classification of differentially expressed proteins via Panther Classification System, based on molecular function. S3 Figure depicts equal loading of secretome samples utilized for Western blot analysis and S4 Figure illustrates the high quality of secretome samples, since the contamination of intracellular proteins (such as tubulin) as shown by Western blot analysis is minor. S5 Figure shows Western blots for HSP90AB in total cell extract and secretome. [file 4180703.f1.pdf]

Supplementary Materials

Cervical cancer cell line secretome highlights the roles of transforming growth factor-beta-induced protein ig-h3, peroxiredoxin-2 and NRF2 on cervical carcinogenesis.

Georgia Kontostathi, Jerome Zoidakis, Manousos Makridakis, Vasiliki Lygirou, George Mermelekas, Theofilos Papadopoulos, Konstantinos Vougas, Alexios Vlamis-Gardikas, Peter Drakakis, Dimitrios Loutradis, Antonia Vlahou, Nicholas P. Anagnou, and Kalliopi I. Pappa

Table Legends

S1 Table. Transition list of endogenous and heavy peptide as exported from skyline and imported in the acquisition method. The mass (m/z) in the first (Q1) and third quadrupole (Q3), the dwell time and the peptide sequence, as well as the charge state and the collision energy of the corresponding fragments are shown.

S2 Table. MRM quantification data. Light to heavy ratio of the peptide peak areas used for NRF2 protein quantification in each biological replicate.

S3 Table. Differentially expressed proteins in secretome analysis of SiHa *vs* HCK1T. Forty-five differentially expressed spots corresponding to 40 proteins in SiHa *vs* HCK1T comparison. The Mann-Whitney test was used for the statistical analysis. A *p* value <0.05 (95% confidence levels) was considered statistically significant.

S4 Table. Differentially expressed proteins in secretome analysis of HeLa *vs* HCK1T. Forty-three differentially expressed spots corresponding to 44 proteins in HeLa *vs* HCK1T comparison. The Mann-Whitney test was used for the statistical analysis. A *p* value <0.05 (95% confidence levels) was considered statistically significant.

S5 Table. Differentially expressed proteins in secretome analysis of C33A *vs* HCK1T. Fifty-three differentially expressed spots corresponding to 41 proteins in C33A *vs* HCK1T comparison. The Mann-Whitney test was used for the statistical analysis. A *p* value <0.05 (95% confidence levels) was considered statistically significant.

S6 Table. Comparison of differentially expressed proteins in secretome analysis between individual comparisons in each cancer cell line *vs* HCK1T. Sixteen proteins are common in all comparisons.

S7 Table. Molecular function of differentially expressed proteins in secretome analysis. Predicted molecular function of differentially expressed proteins in all cancer cell lines *vs* HCK1T, by Panther Classification System (http://www.pantherdb.org/).

S8 Table. Differentially expressed proteins used in IPA. Sixty-seven differentially expressed proteins were used in IPA analysis after they were converted to gene names. Data were submitted to IPA as fold change values (ratios) calculated against the control group (HCK1T). The mean ratio was taken from all identified spots and only proteins with fold change >±2 were taken into account.

S9 Table. IPA results for transcription factors in cancer *vs* HCK1T secretome. IPA results showing the main transcription factors involved in regulation of the differentially expressed proteins in cancer *vs* normal.

S10 Table. Additional number of transcription factors that regulate NRF2 targets. The number of transcription regulators of NRF2 targets are listed.

S1 Table: Transition list

Q1 m/z	Q3 m/z	Dwell time (ms)	Protein	Peptide	Fragment	Charge state	Collision Energy (eV)
701.368	858.439	20	NRF2	LVETTMVPSPEAK (+2) Light	y8	+1	35.7
701.368	727.399	20	NRF2	LVETTMVPSPEAK (+2) Light	y7	+1	35.7
701.368	628.330	20	NRF2	LVETTMVPSPEAK (+2) Light	y6	+1	35.7
701.368	444.245	20	NRF2	LVETTMVPSPEAK (+2) Light	y4	+1	35.7
701.368	314.669	20	NRF2	LVETTMVPSPEAK (+2) Light	y6	+2	35.7

705.375	866.453	20	NRF2	LVETTMVPSPEAK (+2) Heavy	y8	+1	35.7
705.375	735.413	20	NRF2	LVETTMVPSPEAK (+2) Heavy	y7	+1	35.7
705.375	636.344	20	NRF2	LVETTMVPSPEAK (+2) Heavy	y6	+1	35.7
705.375	452.260	20	NRF2	LVETTMVPSPEAK (+2) Heavy	y4	+1	35.7
705.375	318.676	20	NRF2	LVETTMVPSPEAK (+2) Heavy	y6	+2	35.7

S2 Table: Quantification data

Sample	L/H ratio	Sample	L/H ratio
**SiHa 1**	1.446	**C33A 1**	1.418
**SiHa 2**	1.478	**C33A 2**	1.277
**SiHa 3**	1.027	**C33A 3**	1.987
**HeLa 1**	0.764	**HCK1T 1**	0.960
**HeLa 2**	1.053	**HCK1T 2**	0.843
**HeLa 3**	0.816	**HCKT1T 3**	0.839

S3 Table: Differentially expressed proteins between SiHa *vs* HCK1T cells

Spot	Fold change (Ratio SiHa *vs* HCK1T)	Mean SiHa±sd	Mean HCK1T±sd	Protein name	Entry name	Mascot score	Sequence coverage(%)	Protein MW	p*I*-value	*p* value
**1**	3.89	1600± 2100	420± 500	Complement C1r subcomponent	C1R_HUMAN	81	32	81606	5.8	>0.05
**2**	4.59	930± 870	204± 76	Follistatin-related protein 4	FSTL4_HUMAN	134	45	94291	5.9	>0.05
	4.59	930± 870	204± 76	Neutral alpha-glucosidase AB	GANAB_HUMAN	129	37	107263	5.7	>0.05
	4.59	930± 870	204± 76	Procollagen-lysine,2-oxoglutarate 5-dioxygenase 2	PLOD2_HUMAN	108	41	85373	6.3	>0.05

**3**	0.43	1600± 780	3600± 2200	Glucosidase 2 subunit beta	GLU2B_HUMAN	81	34	60357	4.2	>0.05
**4**	12.86	1010± 650	78± 140	Complement C1r subcomponent	C1R_HUMAN	89	29	81606	5.8	<0.05
**5**	12.2	870± 340	71± 64	Gelsolin	GELS_HUMAN	93	31	86043	5.9	<0.05
**6**	0.38	6300± 4000	16000± 17000	78 kDa glucose-regulated protein	GRP78_HUMAN	282	51	72402	4.9	>0.05
**7**	3.12	2980± 860	950± 1400	Heat shock cognate 71 kDa protein	HSP7C_HUMAN	184	45	71082	5.2	>0.05

**8**	3.85	1800± 570	460± 350	Heat shock 70 kDa protein 1A/1B	HSP71_HUMAN	195	59	70294	5.4	<0.05
	3.85	1800± 570	460± 350	Tripeptidyl-peptidase 1	TPP1_HUMAN	68	40	61723	6	<0.05
**9**	32.95	4200± 2000	130± 110	Proprotein convertase subtilisin/kexin type 9	PCSK9_HUMAN	157	26	75722	6.1	<0.05
**10**	4.29	1700± 300	390± 180	Proprotein convertase subtilisin/kexin type 9	PCSK9_HUMAN	104	77	75722	6.1	<0.05

**11**	0.47	3070± 1300	6500± 5800	Protein disulfide-isomerase	PDIA1_HUMAN	256	61	57480	4.6	>0.05
**12**	16.41	8300± 3200	510± 240	Nucleobindin-1	NUCB1_HUMAN	275	60	53846	5	<0.05
**13**	0.17	1100± 400	6400± 2300	Protein disulfide-isomerase A3	PDIA3_HUMAN	145	46	57146	5.9	>0.05
**14**	0.28	1400± 200	5000± 4900	Protein disulfide-isomerase A3	PDIA3_HUMAN	209	55	57146	5.9	>0.05
**15**	6.20	690± 470	110± 76	Keratin, type II cytoskeletal 1	K2C1_HUMAN	69	24	66170	8.8	>0.05

**16**	2.30	1500± 1400	640± 240	Pyruvate kinase isozymes M1/M2	KPYM_HUMAN	216	48	58470	9	>0.05
**17**	0.46	1300± 170	2700± 900	Pyruvate kinase isozymes M1/M2	KPYM_HUMAN	205	52	58470	9	<0.05
**18**	0.07	3100± 1400	41000± 20200	Calreticulin	CALR_HUMAN	202	60	48283	4.1	<0.05
**19**	10.08	21000± 12000	2100± 1300	Carboxypeptidase E	CBPE_HUMAN	190	46	53516	4.9	<0.05

**20**	6.66	2300± 2000	340± 300	Cathepsin D	CATD_HUMAN	116	44	45037	6.1	>0.05
**21**	20.39	6300± 390	310± 170	Cathepsin D	CATD_HUMAN	201	53	45037	6.1	<0.05
**22**	5.85	3600± 2600	620± 600	Cathepsin D	CATD_HUMAN	77	42	45037	6.1	<0.05
**23**	0.27	4900± 3400	19000± 9200	Alpha-enolase	ENOA_HUMAN	289	75	47481	7.7	<0.05
**24**	0.28	11000± 4100	40000± 22000	Actin, cytoplasmic 2	ACTG_HUMAN	178	68	42108	5.2	>0.05
	0.28	11000± 4060	40000± 22000	Actin, cytoplasmic 1	ACTB_HUMAN	166	63	42052	5.2	>0.05

**25**	0.34	2300± 440	6900± 3000	Serpin B5	SPB5_HUMAN	93	60	42530	5.7	<0.05
**26**	0.35	1200± 690	3600± 1600	Serpin B5	SPB5_HUMAN	124	59	42530	5.7	>0.05
**27**	0.23	420± 390	1800± 820	Cathepsin B	CATB_HUMAN	69	39	38766	5.9	>0.05
**28**	0.22	2200± 360	10200± 3100	Fructose-bisphosphate aldolase A	ALDOA_HUMAN	226	73	39851	9.2	<0.05
**29**	11.58	2300± 2900	200± 160	Keratin, type I cytoskeletal 10	K1C10_HUMAN	79	29	59020	5	>0.05

**30**	9.79	930± 230	100± 130	HLA class I histocompatibility antigen, A-24 alpha chain	1A24_HUMAN	90	50	40948	5.9	<0.05
**31**	0.45	5100± 1600	11000± 7800	Glyceraldehyde-3-phosphate dehydrogenase	G3P_HUMAN	62	41	36201	9.3	>0.05
**32**	3.12	880± 460	280± 260	Metalloproteinase inhibitor 1	TIMP1_HUMAN	55	53	23840	9.8	>0.05
**33**	33.38	12000± 5300	360± 310	Phosphoglycerate mutase 1	PGAM1_HUMAN	99	45	28900	6.8	<0.05
	33.38	12000± 5300	360± 310	Metalloproteinase inhibitor 1	TIMP1_HUMAN	75	45	23840	9.8	<0.05
**34**	13.26	11000± 2400	830± 480	Metalloproteinase inhibitor 1	TIMP1_HUMAN	95	54	23840	9.8	<0.05

**35**	0.01	66± 82	12000± 3000	14-3-3 protein sigma	1433S_HUMAN	105	65	27871	4.5	<0.05
**36**	0.15	310± 390	2000± 960	Rho GDP-dissociation inhibitor 1	GDIR1_HUMAN	116	44	23250	4.9	>0.05
**37**	0.05	280± 170	5300± 1700	Heat shock protein beta-1	HSPB1_HUMAN	145	71	22826	6	<0.05
**38**	0.29	670± 350	2300± 1200	Phosphoglycerate mutase 1	PGAM1_HUMAN	131	73	28900	6.8	>0.05
**39**	0.21	1200± 920	5500± 2000	Triosephosphate isomerase	TPIS_HUMAN	118	69	31057	5.6	<0.05
**40**	0.26	890± 320	3500± 1600	Glutathione S-transferase P	GSTP1_HUMAN	137	69	23569	5.3	<0.05

**41**	10.62	9600± 2300	900± 510	Metalloproteinase inhibitor 2	TIMP2_HUMAN	106	51	25067	8.8	<0.05
**42**	0.16	220± 110	1400± 540	Superoxide dismutase [Mn], mitochondrial	SODM_HUMAN	53	40	24878	9.1	<0.05
**43**	0.45	1500± 810	3200± 1100	Transgelin-2	TAGL2_HUMAN	94	62	22548	9.3	<0.05
**44**	21.56	1700± 1100	78± 88	Epididymal secretory protein E1	NPC2_HUMAN	77	50	16902	9	>0.05
**45**	2.47	2200± 620	890± 510	Peptidyl-prolyl cis-trans isomerase A	PPIA_HUMAN	78	53	18229	9	>0.05

S4 Table: Differentially expressed proteins between HeLa *vs* HCK1T cells

Spot	Fold change (Ratio HeLa *vs* HCK1T)	Mean HeLa±sd	Mean HCK1T±sd	Protein name	Entry name	Mascot score	Sequence coverage	Protein MW	p*I*-value	*p* value
**1**	11.2	2300± 1100	204± 76	Follistatin-related protein 4	FSTL4_HUMAN	134	45	94291	5.9	<0.05
	11.2	2300± 1100	204± 76	Neutral alpha-glucosidase AB	GANAB_HUMAN	129	37	107263	5.7	<0.05
	11.2	2300± 1100	204± 76	Procollagen-lysine, 2-oxoglutarate 5-dioxygenase 2	PLOD2_HUMAN	108	41	85373	6.3	<0.05
**2**	0.23	820± 1100	3600± 2200	Glucosidase 2 subunit beta	GLU2B_HUMAN	81	34	60357	4.2	>0.05

**3**	0.4	1200± 430	3100± 1100	Laminin subunit gamma-2	LAMC2_HUMAN	192	25	134769	5.8	>0.05
**4**	11.14	3200± 1000	290± 320	Heat shock protein HSP 90-beta	HS90B_HUMAN	95	35	83554	4.8	<0.05
**5**	3.11	240± 220	80± 140	Complement C1r subcomponent	C1R_HUMAN	89	29	81606	5.8	>0.05
**6**	2.92	210± 180	71± 64	Gelsolin	GELS_HUMAN	93	31	86043	5.9	>0.05
**7**	0.39	6400± 1400	16000± 17000	78 kDa glucose-regulated protein	GRP78_HUMAN	282	51	72402	4.9	>0.05

**8**	2.81	2700± 2400	950± 1400	Heat shock cognate 71 kDa protein	HSP7C_HUMAN	184	45	71082	5.2	>0.05
**9**	6.25	2900± 680	460± 350	Heat shock 70 kDa protein 1A/1B	HSP71_HUMAN	195	59	70294	5.4	<0.05
	6.25	2900± 680	460± 350	Tripeptidyl-peptidase 1	TPP1_HUMAN	68	40	61723	6	<0.05
**10**	45.34	5800± 2000	128± 110	Proprotein convertase subtilisin/kexin type 9	PCSK9_HUMAN	157	26	75722	6.1	<0.05
**11**	8.03	3200± 990	390± 180	Proprotein convertase subtilisin/kexin type 9	PCSK9_HUMAN	104	77	75722	6.1	<0.05

**12**	7.23	3600± 810	500± 80	Stress-induced-phosphoprotein 1	STIP1_HUMAN	141	56	63227	6.4	<0.05
**13**	45.15	5100± 5100	110± 170	Transforming growth factor-beta-induced protein ig-h3	BGH3_HUMAN	99	37	75261	8.6	<0.05
**14**	7.86	3300± 190	420± 250	Transketolase	TKT_HUMAN	126	47	68519	8.5	<0.05
**15**	10.26	2600± 820	260± 210	Transketolase	TKT_HUMAN	85	35	68519	8.5	<0.05
**16**	5.51	2800± 1100	510± 240	Nucleobindin-1	NUCB1_HUMAN	275	60	53846	5	<0.05
**17**	2.21	1200± 860	540± 630	Dipeptidyl peptidase 2	DPP2_HUMAN	53	19	54763	5.9	>0.05

**18**	3.39	970± 870	290± 380	T-complex protein 1 subunit theta	TCPQ_HUMAN	152	48	60153	5.3	>0.05
**19**	5.93	660± 320	110± 76	Keratin, type II cytoskeletal 1	K2C1_HUMAN	134	24	66170	8.8	>0.05
**20**	3.96	2500± 2100	640± 240	Pyruvate kinase isozymes M1/M2	KPYM_HUMAN	216	48	58470	9	<0.05
**21**	0.24	1600± 810	6500± 5800	Protein disulfide-isomerase	PDIA1_HUMAN	256	61	57480	4.6	>0.05
**22**	0.23	9400± 3700	41000± 20000	Calreticulin	CALR_HUMAN	202	60	48283	4.1	<0.05

**23**	4.71	9700± 4600	2050± 1300	Carboxypeptidase E	CBPE_HUMAN	190	46	53516	4.9	<0.05
**24**	0.25	750± 970	3100± 1700	Protein disulfide-isomerase A6	PDIA6_HUMAN	118	46	48490	4.8	>0.05
	0.25	750± 970	3100± 1700	ATP synthase subunit beta, mitochondrial	ATPB_HUMAN	72	34	56525	5.1	>0.05
**25**	2.54	1300± 310	510± 200	Fascin	FSCN1_HUMAN	184	55	55123	7	<0.05
**26**	11.24	3800± 2800	340± 300	Cathepsin D	CATD_HUMAN	116	44	45037	6.1	<0.05

**27**	9.79	3030± 94	310± 170	Cathepsin D	CATD_HUMAN	201	53	45037	6.1	<0.05
**28**	9.35	5800± 2100	620± 600	Cathepsin D	CATD_HUMAN	77	42	45037	6.1	>0.05
**29**	0.34	6400± 4400	19000± 9200	Alpha-enolase	ENOA_HUMAN	289	75	47481	7.7	>0.05
**30**	0.20	8100± 1500	40000± 22000	Actin, cytoplasmic 2	ACTG_HUMAN	178	68	42108	5.2	>0.05
	0.20	8100± 1500	40000± 22000	Actin, cytoplasmic 1	ACTB_HUMAN	166	63	42052	5.2	>0.05

**31**	2.21	8200± 3700	3700± 2500	Cathepsin Z	CATZ_HUMAN	59	44	34530	6.9	>0.05
**32**	2.37	1600± 460	660± 140	Phosphoglycerate kinase 1	PGK1_HUMAN	81	40	44985	9.2	>0.05
**33**	48.84	9600± 3500	200± 150	Keratin, type I cytoskeletal 10	K1C10_HUMAN	79	29	59020	5	>0.05
**34**	0.29	3000± 2500	10000± 3100	Fructose-bisphosphate aldolase A	ALDOA_HUMAN	226	73	39851	9.2	<0.05
**35**	15.53	1500± 160	100± 130	HLA class I histocompatibility antigen, A-24 alpha chain	1A24_HUMAN	90	50	40948	5.9	>0.05

**36**	4.12	4500± 1700	1100± 850	Heterogeneous nuclear ribonucleoprotein A1	ROA1_HUMAN	114	43	38837.00	9.6	<0.05
**37**	2.57	920± 250	360± 310	Phosphoglycerate mutase 1	PGAM1_HUMAN	99	45	28900	6.8	<0.05
	2.57	920± 250	360± 3000	Metalloproteinase inhibitor 1	TIMP1_HUMAN	75	45	23840	9.8	<0.05
**38**	0.20	310± 330	1500± 700	Keratin, type I cytoskeletal 10	K1C10_HUMAN	95	43	59020	5	<0.05
**39**	0.11	560± 310	5300± 1700	Heat shock protein beta-1	HSPB1_HUMAN	145	71	22826	6	<0.05

**40**	0.23	1300± 1100	5500± 2000	Triosephosphate isomerase	TPIS_HUMAN	143	69	31057	5.6	<0.05
**41**	0.11	90± 84	830± 310	Protein DJ-1	PARK7_HUMAN	114	83	20050	6.4	<0.05
**42**	8.04	7300± 1900	900± 510	Metalloproteinase inhibitor 2	TIMP2_HUMAN	106	51	25067	8.8	<0.05
**43**	0.37	2600± 1600	7200± 1400	Peptidyl-prolyl cis-trans isomerase A	PPIA_HUMAN	116	70	18229	9	>0.05

S5 Table: Differentially expressed proteins between C33A *vs* HCK1T cells

Spot	Fold change (Ratio C33A *vs* HCK1T)	Mean C33A±sd	Mean HCK1T±sd	Protein name	Entry name	Mascot score	Sequence coverage	Protein MW	p*I*-value	*p value*
**1**	0.16	68± 59	430± 180	Fibronectin	FINC_HUMAN	147	21	266052	5.4	>0.05
**2**	0.30	180± 190	590± 390	Fibronectin	FINC_HUMAN	257	34	266052	5.4	>0.05
**3**	0.21	130± 62	610± 400	Fibronectin	FINC_HUMAN	93	22	266052	5.4	<0.05
**4**	0.10	46± 24	460± 540	Fibronectin	FINC_HUMAN	367	40	266052	5.4	<0.05

**5**	0.23	110± 83	490± 490	Fibronectin	FINC_HUMAN	239	32	266052	5.4	>0.05
**6**	0.23	220± 220	970± 660	Fibronectin	FINC_HUMAN	274	34	266052	5.4	>0.05
**7**	0.25	200± 98	810± 490	Fibronectin	FINC_HUMAN	328	33	266052	5.4	>0.05
**8**	0.35	370± 180	1060± 1000	Fibronectin	FINC_HUMAN	165	26	266052	5.4	>0.05
**9**	5.15	2800± 2800	540± 610	Heat shock 70 kDa protein 4	HSP74_HUMAN	129	49	95127	5	>0.05
**10**	0.40	1400± 380	3600± 2200	Glucosidase 2 subunit beta	GLU2B_HUMAN	81	34	60357	4.2	>0.05

**11**	0.47	1400± 790	3050± 1100	Laminin subunit gamma-2	LAMC2_HUMAN	192	25	134769	5.8	>0.05
**12**	2.21	2700± 1200	1200± 370	Transitional endoplasmic reticulum ATPase	TERA_HUMAN	162	49	89950	5	<0.05
**13**	16.22	4700± 1300	290± 320	Heat shock protein HSP 90-beta	HS90B_HUMAN	95	35	83554	4.8	<0.05
**14**	0.23	3700± 1960	16400± 17000	78 kDa glucose-regulated protein	GRP78_HUMAN	282	51	72402	4.9	>0.05
**15**	4.53	4300± 950	950± 1400	Heat shock cognate 71 kDa protein	HSP7C_HUMAN	184	45	71082	5.2	>0.05

**16**	6.44	2970± 510	460± 350	Heat shock 70 kDa protein 1A/1B	HSP71_HUMAN	195	59	70294	5.4	>0.05
	6.44	2970± 510	460± 350	Tripeptidyl-peptidase 1	TPP1_HUMAN	68	40	61723	6	>0.05
**17**	9	1200± 1040	128± 105	Proprotein convertase subtilisin/kexin type 9	PCSK9_HUMAN	157	26	75722	6.1	>0.05
**18**	3.01	1200± 1100	390± 180	Proprotein convertase subtilisin/kexin type 9	PCSK9_HUMAN	104	77	75722	6.1	>0.05
**19**	6.74	3400± 830	500± 80	Stress-induced-phospho-protein 1	STIP1_HUMAN	141	56	63227	6.4	<0.05

**20**	2.09	880± 610	420± 250	Transketolase	TKT_HUMAN	126	47	68519	8.5	>0.05
**21**	5.74	1500± 1100	260± 210	Transketolase	TKT_HUMAN	85	35	68519	8.5	>0.05
**22**	15.69	7900± 3200	505± 240	Nucleobindin-1	NUCB1_HUMAN	275	60	53846	5	<0.05
**23**	9.67	2800± 920	290± 380	T-complex protein 1 subunit theta	TCPQ_HUMAN	152	48	60153	5.3	<0.05
**24**	2.17	1400± 1100	640± 240	Pyruvate kinase isozymes M1/M2	KPYM_HUMAN	216	48	58470	9	>0.05
**25**	0.07	2900± 1700	41300± 20200	Calreticulin	CALR_HUMAN	202	60	48283	4.1	<0.05

**26**	0.27	1800± 540	6500± 5800	Protein disulfide-isomerase	PDIA1_HUMAN	256	61	57480	4.6	>0.05
**27**	6.95	14000± 4800	2050± 1300	Carboxy-peptidase E	CBPE_HUMAN	190	46	53516	4.9	<0.05
**28**	0.46	3000± 1700	6400± 2300	Protein disulfide-isomerase A3	PDIA3_HUMAN	145	46	57146	5.9	>0.05
**29**	0.04	180± 150	5000± 4900	Protein disulfide-isomerase A3	PDIA3_HUMAN	209	55	57146	5.9	<0.05
**30**	11.52	1800± 320	150± 100	Alpha-enolase	ENOA_HUMAN	223	79	47481	7.7	<0.05

**31**	2.88	890± 340	310± 170	Cathepsin D	CATD_HUMAN	116	53	45037	6.1	<0.05
**32**	7.86	6100± 1000	780± 330	Elongation factor 1-gamma	EF1G_HUMAN	126	49	50429	6.3	<0.05
**33**	2.39	14000± 5100	5900± 1600	Elongation factor 1-alpha 1	EF1A1_HUMAN	63	40	50451	9.7	>0.05
**34**	0.25	1700± 1300	6900± 3000	Serpin B5	SPB5_HUMAN	93	60	42530	5.7	<0.05
**35**	0.09	320± 160	3600± 1600	Serpin B5	SPB5_HUMAN	124	59	42530	5.7	>0.05

**36**	0.15	270 190	1800± 820	Cathepsin B	CATB_HUMAN	69	39	38766	5.9	<0.05
**37**	3.31	1200± 420	360± 150	Poly(rC)-binding protein 1	PCBP1_HUMAN	87	55	37987	6.8	<0.05
**38**	16.74	1600± 410	95± 130	60S acidic ribosomal protein P0	RLA0_HUMAN	81	46	34423	5.6	<0.05
**39**	6.80	7600± 1100	1100± 660	L-lactate dehydro-genase B chain	LDHB_HUMAN	116	55	36900	5.7	<0.05
**40**	9.45	10200± 3900	1100± 850	Hetero-geneous nuclear ribonucleo-protein A1	ROA1_HUMAN	114	43	38837.0 0	9.6	>0.05

**41**	0.14	1700± 1300	12000± 3000	14-3-3 protein sigma	1433S_HUMAN	105	65	27871	4.5	<0.05
**42**	0.21	1100± 760	5300± 1700	Heat shock protein beta-1	HSPB1_HUMAN	145	71	22826	6	<0.05
**43**	9.66	6700± 1600	690± 450	Peroxiredoxin-6	PRDX6_HUMAN	123	64	25133	6	<0.05
**44**	3.06	3600± 1000	1200± 320	Triose-phosphate isomerase	TPIS_HUMAN	98	62	31057	5.6	>0.05
**45**	0.25	860± 780	3500± 1600	Glutathione S-transferase P	GSTP1_HUMAN	137	69	23569	5.3	<0.05

**46**	2.52	2500± 170	1000± 470	Peroxiredoxin-2	PRDX2_HUMAN	117	55	22049	5.6	>0.05
**47**	0.02	26± 13.67	1400± 540	Superoxide dismutase [Mn], mitochondrial	SODM_HUMAN	53	40	24878	9.1	<0.05
**48**	2.70	5600± 940	2100± 820	Peroxiredoxin-1	PRDX1_HUMAN	149	79	22324	9.2	>0.05
**49**	2.08	1300± 330	610± 450	Nucleoside diphosphate kinase A	NDKA_HUMAN	64	53	17309	5.8	>0.05
**50**	13.37	1300± 1400	99± 120	Cofilin-1	COF1_HUMAN	86	65	18719	9.1	>0.05

**51**	0.09	170± 150	1800± 800	Peptidyl-prolyl cis-trans isomerase A	PPIA_HUMAN	58	39	18229	9	<0.05
**52**	2.02	1800± 2000	890± 500	Peptidyl-prolyl cis-trans isomerase A	PPIA_HUMAN	78	53	18229	9	>0.05
**53**	0.31	2300± 2400	7200± 1400	Peptidyl-prolyl cis-trans isomerase A	PPIA_HUMAN	116	70	18229	9	>0.05

S6 Table: Comparisons of differentially expressed proteins between individual comparisons in each cancer cell line *vs* HCK1T

Comparison	Number of common proteins between individual comparisons	Entry names	Protein names
**SiHa *vs* HCK1T, HeLa *vs* HCK1T, C33A *vs* HCK1T**	16	PPIA_HUMAN	Peptidyl-prolyl cis-trans isomerase A
		ENOA_HUMAN	Alpha-enolase
		GLU2B_HUMAN	Glucosidase 2 subunit beta
		HSPB1_HUMAN	Heat shock protein beta-1
		TPIS_HUMAN	Triosephosphate isomerase
		PDIA1_HUMAN	Protein disulfide-isomerase
		KPYM_HUMAN	Pyruvate kinase PKM
		NUCB1_HUMAN	Nucleobindin-1
		PCSK9_HUMAN	Proprotein convertase subtilisin/kexin type 9
		GRP78_HUMAN	78 kDa glucose-regulated protein
		TPP1_HUMAN	Tripeptidyl-peptidase 1
		HSP7C_HUMAN	
		CBPE_HUMAN	
		CATD_HUMAN	
		HSP71_HUMAN	
		CALR_HUMAN	

**SiHa *vs* HCK1T, HeLa *vs* HCK1T**	14	PLOD2_HUMAN	Procollagen-lysine,2-oxoglutarate 5-dioxygenase 2
		GELS_HUMAN	Gelsolin
		K1C10_HUMAN	Keratin, type I cytoskeletal 10
		FSTL4_HUMAN	Follistatin-related protein 4
		GANAB_HUMAN	Neutral alpha-glucosidase AB
		TIMP2_HUMAN	Metalloproteinase inhibitor 2
		ACTB_HUMAN	Actin, cytoplasmic 1
		1A24_HUMAN	HLA class I histocompatibility antigen, A-24 alpha chain
		ACTG_HUMAN	Actin, cytoplasmic 2
		PGAM1_HUMAN	Phosphoglycerate mutase 1
		ALDOA_HUMAN	Fructose-bisphosphate aldolase A
		C1R_HUMAN	Complement C1r subcomponent
		K2C1_HUMAN	Keratin, type II cytoskeletal 1
		TIMP1_HUMAN	Metalloproteinase inhibitor 1

**SiHa *vs* HCK1T, C33A *vs* HCK1T**	6	GSTP1_HUMAN	Glutathione S-transferase P
		SPB5_HUMAN	Serpin B5
		CATB_HUMAN	Cathepsin B
		1433S_HUMAN	14-3-3 protein sigma
		SODM_HUMAN	Superoxide dismutase [Mn], mitochondrial
		PDIA3_HUMAN	Protein disulfide-isomerase A3
**HeLa *vs* HCK1T, C33A *vs* HCK1T**	6	LAMC2_HUMAN	Laminin subunit gamma-2
		ROA1_HUMAN	Heterogeneous nuclear ribonucleoprotein A1
		HS90B_HUMAN	Heat shock protein HSP 90-beta
		TCPQ_HUMAN	T-complex protein 1 subunit theta
		STIP1_HUMAN	Stress-induced-phosphoprotein 1
		TKT_HUMAN	Transketolase

Comparison	Number of unique proteins in individual comparisons	Entry names	Protein names
**SiHa *vs* HCK1T**	4	TAGL2_HUMAN	Transgelin-2
		G3P_HUMAN	Glyceraldehyde-3-phosphate dehydrogenase
		NPC2_HUMAN	Epididymal secretory protein E1
		GDIR1_HUMAN	Rho GDP-dissociation inhibitor 1
**HeLa *vs* HCK1T**	8	FSCN1_HUMAN	Fascin
		DPP2_HUMAN	Dipeptidyl peptidase 2
		PDIA6_HUMAN	Protein disulfide-isomerase A6
		BGH3_HUMAN	Transforming growth factor-beta-induced protein ig-h3
		ATPB_HUMAN	ATP synthase subunit beta, mitochondrial
		PARK7_HUMAN	Protein deglycase DJ-1
		CATZ_HUMAN	Cathepsin Z
		PGK1_HUMAN	Phosphoglycerate kinase 1

**C33A *vs* HCK1T**	13	PCBP1_HUMAN	Poly(rC)-binding protein 1
		COF1_HUMAN	Cofilin-1
		TERA_HUMAN	Transitional endoplasmic reticulum ATPase
		EF1G_HUMAN	Elongation factor 1-gamma
		EF1A1_HUMAN	Elongation factor 1-alpha 1
		PRDX1_HUMAN	Peroxiredoxin-1
		HSP74_HUMAN	Heat shock 70 kDa protein 4
		FINC_HUMAN	Fibronectin
		LDHB_HUMAN	L-lactate dehydrogenase B chain
		PRDX6_HUMAN	Peroxiredoxin-6
		RLA0_HUMAN	60S acidic ribosomal protein P0
		NDKA_HUMAN	Nucleoside diphosphate kinase A
		PRDX2_HUMAN	Peroxiredoxin-2

S7 Table. Molecular function of differentially expressed proteins

Entry name	Protein name	Molecular function
**1433S_HUMAN**	14-3-3 protein sigma	binding, enzyme regulator activity
**1A24_HUMAN**	HLA class I histocompatibility antigen, A-24 alpha chain	receptor activity
**ACTB_HUMAN**	Actin, cytoplasmic 1	structural molecule activity
**ACTG_HUMAN**	Actin, cytoplasmic 2	structural molecule activity
**ALDOA_HUMAN**	Fructose-bisphosphate aldolase A	binding, catalytic
**C1R_HUMAN**	Complement C1r subcomponent	binding, catalytic
**CALR_HUMAN**	Calreticulin	binding
**CATB_HUMAN**	Cathepsin B	catalytic
**CATD_HUMAN**	Cathepsin D	catalytic
**CBPE_HUMAN**	Carboxypeptidase E	catalytic

**ENOA_HUMAN**	Alpha-enolase	catalytic
**FSTL4_HUMAN**	Follistatin-related protein 4	binding
**G3P_HUMAN**	Glyceraldehyde-3-phosphate dehydrogenase	catalytic
**GANAB_HUMAN**	Neutral alpha-glucosidase AB	catalytic
**GDIR1_HUMAN**	Rho GDP-dissociation inhibitor 1	binding, catalytic, enzyme regulator activity
**GELS_HUMAN**	Gelsolin	binding, structural molecule activity
**GLU2B_HUMAN**	Glucosidase 2 subunit beta	binding, catalytic, enzyme regulator activity
**GRP78_HUMAN**	78 kDa glucose-regulated protein	binding,catalytic
**GSTP1_HUMAN**	Glutathione S-transferase P	binding,catalytic
**HS71A_HUMAN**	Heat shock 70 kDa protein 1A	binding, catalytic

**HSP7C_HUMAN**	Heat shock cognate 71 kDa protein	binding, catalytic
**HSPB1_HUMAN**	Heat shock protein beta-1	structural molecule activity
**K1C10_HUMAN**	Keratin, type I cytoskeletal 10	structural molecule activity
**K2C1_HUMAN**	Keratin, type II cytoskeletal 1	structural molecule activity
**KPYM_HUMAN**	Pyruvate kinase PKM	binding, catalytic
**NPC2_HUMAN**	Epididymal secretory protein E1	binding
**NUCB1_HUMAN**	Nucleobindin-1	binding
**PCSK9_HUMAN**	Proprotein convertase subtilisin/kexin type 9	catalytic
**PDIA1_HUMAN**	Protein disulfide-isomerase	catalytic
**PDIA3_HUMAN**	Protein disulfide-isomerase A3	catalytic

**PGAM1_HUMAN**	Phosphoglycerate mutase 1	binding, catalytic
**PLOD2_HUMAN**	Procollagen-lysine, 2-oxoglutarate 5-dioxygenase 2	catalytic
**PPIA_HUMAN**	Peptidyl-prolyl cis-trans isomerase A	catalytic
**SODM_HUMAN**	Superoxide dismutase [Mn], mitochondrial	catalytic
**SPB5_HUMAN**	Serpin B5	catalytic, enzyme regulator activity
**TAGL2_HUMAN**	Transgelin-2	binding, structural molecule activity
**TIMP1_HUMAN**	Metalloproteinase inhibitor 1	binding, catalytic, enzyme regulator activity
**TIMP2_HUMAN**	Metalloproteinase inhibitor 2	binding, catalytic, enzyme regulator activity
**TPIS_HUMAN**	Triosephosphate isomerase	catalytic
**TPP1_HUMAN**	Tripeptidyl-peptidase 1	catalytic

**ATPB_HUMAN**	ATP synthase subunit beta, mitochondrial	binding, catalytic, receptor activity, transporter activity
**BGH3_HUMAN**	Transforming growth factor-beta-induced protein ig-h3	binding
**CATZ_HUMAN**	Cathepsin Z	catalytic
**DPP2_HUMAN**	Dipeptidyl peptidase 2	catalytic
**FSCN1_HUMAN**	Fascin	binding , structural molecule activity
**HS90B_HUMAN**	Heat shock protein HSP 90-beta	binding
**LAMC2_HUMAN**	Laminin subunit gamma-2	receptor activity
**PARK7_HUMAN**	Protein deglycase DJ-1	binding, catalytic, nucleid acid binding, transcription factor activity
**PDIA6_HUMAN**	Protein disulfide-isomerase A6	catalytic
**PGK1_HUMAN**	Phosphoglycerate kinase 1	catalytic

**ROA1_HUMAN**	Heterogeneous nuclear ribonucleoprotein A1	Binding, catalytic, structural molecule activity
**STIP1_HUMAN**	Stress-induced-phosphoprotein 1	binding
**TCPQ_HUMAN**	T-complex protein 1 subunit theta	binding, catalytic
**TKT_HUMAN**	Transketolase	catalytic
**COF1_HUMAN**	Cofilin-1	binding
**EF1A1_HUMAN**	Elongation factor 1-alpha 1	binding, catalytic, translation regulator activity
**EF1G_HUMAN**	Elongation factor 1-gamma	binding, catalytic, structural molecule activity, translation regulator activity
**FINC_HUMAN**	Fibronectin	binding
**HSP74_HUMAN**	Heat shock 70 kDa protein 4	binding
**LDHB_HUMAN**	L-lactate dehydrogenase B chain	catalytic

**NDKA_HUMAN**	Nucleoside diphosphate kinase A	catalytic
**PCBP1_HUMAN**	Poly(rC)-binding protein 1	binding, catalytic
**PRDX1_HUMAN**	Peroxiredoxin-1	catalytic, antioxidant
**PRDX2_HUMAN**	Peroxiredoxin-2	catalytic, antioxidant
**PRDX6_HUMAN**	Peroxiredoxin-6	catalytic, antioxidant
**RLA0_HUMAN**	60S acidic ribosomal protein P0	binding, structural molecule activity
**TERA_HUMAN**	Transitional endoplasmic reticulum ATPase	binding,catalytic

S8 Table. Differentially expressed proteins used in IPA

Protein name	Entry name	Gene name	Fold change
**Actin, cytoplasmic 1**	ACTB_HUMAN	ACTB	-4.17
**Actin, cytoplasmic 2**	ACTG_HUMAN	ACTG1	-4.17
**Fructose-bisphosphate aldolase A**	ALDOA_HUMAN	ALDOA	-3.92
**Rho GDP-dissociation inhibitor 1**	GDIR1_HUMAN	ARHGDIA	-6.67
**ATP synthase subunit beta, mitochondrial**	ATPB_HUMAN	ATP5B	-4.00
**Complement C1r subcomponent**	C1R_HUMAN	C1R	5.74
**Calreticulin**	CALR_HUMAN	CALR	-8.11
**T-complex protein 1 subunit theta**	TCPQ_HUMAN	CCT8	6.53
**Cofilin-1**	COF1_HUMAN	CFL1	13.37
**Carboxypeptidase E**	CBPE_HUMAN	CPE	7.25
**Cathepsin B**	CATB_HUMAN	CTSB	-5.26
**Cathepsin D**	CATD_HUMAN	CTSD	7.99
**Cathepsin Z**	CATZ_HUMAN	CTSZ	2.21

**Dipeptidyl peptidase 2**	DPP2_HUMAN	DPP7	2.21
**Elongation factor 1-alpha 1**	EF1A1_HUMAN	EEF1A1	2.39
**Elongation factor 1-gamma**	EF1G_HUMAN	EEF1G	7.86
**Alpha-enolase**	ENOA_HUMAN	ENO1	-2.86
**Fibronectin**	FINC_HUMAN	FN1	-4.37
**Fascin**	FSCN1_HUMAN	FSCN1	2.54
**Follistatin-related protein 4**	FSTL4_HUMAN	FSTL4	7.90
**Neutral alpha-glucosidase AB**	GANAB_HUMAN	GANAB	7.90
**Glyceraldehyde-3-phosphate dehydrogenase**	G3P_HUMAN	GAPDH	-2.22
**Gelsolin**	GELS_HUMAN	GSN	7.56
**Glutathione S-transferase P**	GSTP1_HUMAN	GSTP1	-3.92
**HLA class I histocompatibility antigen, A-24 alpha chain**	1A24_HUMAN	HLA-A	12.66
**Heterogeneous nuclear ribonucleoprotein A1**	ROA1_HUMAN	HNRNPA1	6.79
**Heat shock protein HSP 90-beta**	HS90B_HUMAN	HSP90AB1	13.68
**Heat shock 70 kDa protein 1B**	HS71B_HUMAN	HSPA1B	5.51
**Heat shock 70 kDa protein 4**	HSP74_HUMAN	HSPA4	5.15

**78 kDa glucose-regulated protein**	GRP78_HUMAN	HSPA5	-3.33
**Heat shock cognate 71 kDa protein**	HSP7C_HUMAN	HSPA8	3.49
**Heat shock protein beta-1**	HSPB1_HUMAN	HSPB1	-8.11
**Keratin, type II cytoskeletal 1**	K2C1_HUMAN	KRT1	6.06
**Keratin, type I cytoskeletal 10**	K1C10_HUMAN	KRT10	8.02
**Laminin subunit gamma-2**	LAMC2_HUMAN	LAMC2	-2.30
**L-lactate dehydrogenase B chain**	LDHB_HUMAN	LDHB	6.80
**Nucleoside diphosphate kinase A**	NDKA_HUMAN	NME1	2.08
**Epididymal secretory protein E1**	NPC2_HUMAN	NPC2	21.56
**Nucleobindin-1**	NUCB1_HUMAN	NUCB1	12.54
**Protein disulfide-isomerase**	PDIA1_HUMAN	P4HB	-3.06
**Protein deglycase DJ-1**	PARK7_HUMAN	PARK7	-9.09
**Poly(rC)-binding protein 1**	PCBP1_HUMAN	PCBP1	3.31

**Proprotein convertase subtilisin/kexin type 9**	PCSK9_HUMAN	PCSK9	17.10
**Protein disulfide-isomerase A3**	PDIA3_HUMAN	PDIA3	-4.21
**Protein disulfide-isomerase A6**	PDIA6_HUMAN	PDIA6	-4.00
**Phosphoglycerate mutase 1**	PGAM1_HUMAN	PGAM1	3.64
**Phosphoglycerate kinase 1**	PGK1_HUMAN	PGK1	2.37
**Pyruvate kinase PKM**	KPYM_HUMAN	PKM	3.07
**Procollagen-lysine, 2-oxoglutarate 5-dioxygenase 2**	PLOD2_HUMAN	PLOD2	7.90
**Peroxiredoxin-1**	PRDX1_HUMAN	PRDX1	2.70
**Peroxiredoxin-2**	PRDX2_HUMAN	PRDX2	2.52
**Peroxiredoxin-6**	PRDX6_HUMAN	PRDX6	9.66
**Glucosidase 2 subunit beta**	GLU2B_HUMAN	PRKCSH	-2.83
**60S acidic ribosomal protein P0**	RLA0_HUMAN	RPLP0	16.74
**Serpin B5**	SPB5_HUMAN	SERPINB5	-3.88
**14-3-3 protein sigma**	1433S_HUMAN	SFN	-13.33
**Superoxide dismutase [Mn], mitochondrial**	SODM_HUMAN	SOD2	-11.11

**Stress-induced-phosphoprotein 1**	STIP1_HUMAN	STIP1	6.99
**Transgelin-2**	TAGL2_HUMAN	TAGLN2	-2.22
**Transforming growth factor-beta-induced protein ig-h3**	BGH3_HUMAN	TGFBI	45.15
**Metalloproteinase inhibitor 1**	TIMP1_HUMAN	TIMP1	9.58
**Metalloproteinase inhibitor 2**	TIMP2_HUMAN	TIMP2	9.33
**Transketolase**	TKT_HUMAN	TKT	6.49
**Triosephosphate isomerase**	TPIS_HUMAN	TPI1	-2.52
**Tripeptidyl-peptidase 1**	TPP1_HUMAN	TPP1	5.51
**Transitional endoplasmic reticulum ATPase**	TERA_HUMAN	VCP	2.21

S9 Table. IPA results for transcription factors in cancer *vs* normal

Transcription Factor	Activation z-score	*p* value of overlap	Target molecules in dataset
**TP53**	-1.792	2.5×10^{-15}	ACTB,CTSB,CTSD,FN1,GAPDH,GSN,GSTP1, HSP90AB1,HSPA1A/HSPA1B,HSPA8,HSPB1, NME1,P4HB,PARK7,PDIA6,PLOD2,PRDX2, PRDX6,SERPINB5,SFN,SOD2,STIP1,TAGLN2, TGFBI,TIMP2,TPP1,
**MYC**	0.769	2.74×10^{-15}	ACTB,ALDOA,CTSB,CTSD,ENO1,FN1,GAPDH ,HLA-A,HNRNPA1,HSPB1,LDHB,NME1,NUCB1,PGAM1,PGK1,PKM,PRDX2,SOD2,TAGLN2,TIMP1, TIMP2,TKT,TPI1,
**MYCN**	0.428	7.23×10^{-15}	ACTB,ACTG1,ALDOA,EEF1A1,EEF1G,FN1,GAPDH,HLA-A,HSP90AB1,NME1,NUCB1,RPLP0,TIMP2, TPI1,
**NFE2L2**	0.033	3.11×10^{-13}	ACTG1,ALDOA,CTSD,FN1,GSTP1,HSP90AB1, PCBP1,PDIA3,PDIA6,PRDX1,RPLP0,SOD2, STIP1,TPI1,VCP

S10 Table. Additional number of transcription factors that regulate NRF2 targets

Targets	Number of TFs
**ACTG1**	5
**ALDOA**	5
**CTSD**	18
**FN1**	44
**GSTP1**	12
**HSP90AB1**	6
**PCBP1**	1
**PDIA3**	2
**PDIA6**	3
**PRDX1**	6
**RPLP0**	7
**SOD2**	28
**STIP1**	4
**TPI1**	7
**VCP**	3

Figure Legends

S1 Fig. Molecular function analysis of differentially expressed proteins identified in the secretome of cancer cell lines compared to HCK1T, as predicted by Panther Classification System (http://www.pantherdb.org/). Sixty-seven proteins were found to be differentially expressed in all three cancer cell lines *vs* HCK1T, and were classified according to their molecular function. The majority of proteins displayed catalytic activity (41.6%), while 32.4% displayed binding activity and 11.1% structural molecule activity.

S2 Fig. Molecular function analysis of differentially expressed proteins identified in the secretome of each cancer cell line compared to HCK1T, as predicted by Panther Classification System (http://www.pantherdb.org/). (A) SiHa *vs* HCK1T, (B) HeLa *vs* HCK1T and (C) C33A *vs* HCK1T. The proteins were classified according to their molecular function. The majority of proteins display catalytic and binding activity, followed by structural molecule activity.

S3 Fig. Representative SDS PAGE gel confirming the equal loading of the secretome samples used for Western blot analysis. Ten µg of secretome of each cell line was loaded and analyzed in precast gradient (4-12%) gels.

S4 Fig. Western blot analysis for tubulin in cell lysates and their corresponding secretomes, providing proof that peroxiredoxin-2 is genuinely secreted and is not a product of cell lysis. Cells were incubated with SFM (Serum-Free Medium) for 24 h after which CM (conditioned medium) was collected. The intensity of tubulin (a marker of intracellular protein contamination of secretome due to extensive cell lysis) in secretome samples deriving from cells incubated with SFM is negligible compared to the corresponding total cell extracts. Representative images of two biological replicates are presented.

S5 Fig. Validation of proteomics and bioinformatics results by Western blot analysis. Western blot analysis for NRF2 target indicated in Fig.4A (HSP90AB1) was performed in C33A *vs* HCK1T, where the upregulation level of NRF2 was higher. Panels A and B represent Western blots for HSP90AB1 in total cell extract and secretome respectively. Representative images of two biological replicates are shown for each cell line. (A) A protein band of 90 kDa corresponding to HSP90AB1 was detected. Immunoblotting for α-tubulin (50 kDa) was applied to ensure equal loading of proteins in each lane. Fold expression of HSP90AB1 was assessed relative to HCK1T. The mean HSP90AB1 value for C33A was 2.3 ± 0.2 ($p < 0.05$) compared to HCK1T. (B) A protein band of 90 kDa corresponding to HSP90AB1 was detected. Fold expression of HSP90AB1 was assessed relative to HCK1T. The mean HSP90AB1 value for C33A was 4.8 ± 1.1 ($p < 0.05$) compared to HCK1T. Graphical representation of densitometry analysis of the results (mean ± SD), is also shown (* $p<0.05$, Student's *t*-test).

S1 Fig.

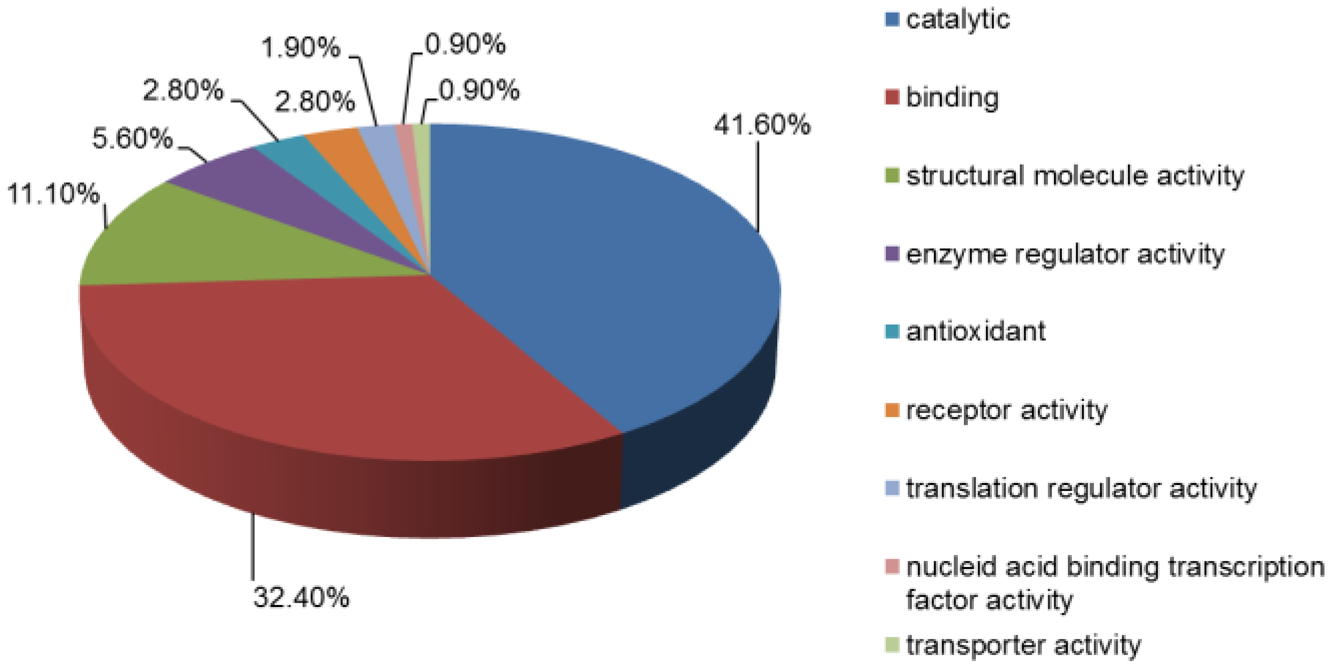
1.90%
0.90%
2.80%
2.80%
0.90%
5.60%
41.60%
11.10%
32.40%
catalytic
binding
structural molecule activity
enzyme regulator activity
antioxidant
receptor activity
translation regulator activity
nucleid acid binding transcription factor activity
transporter activity

S2 Fig.

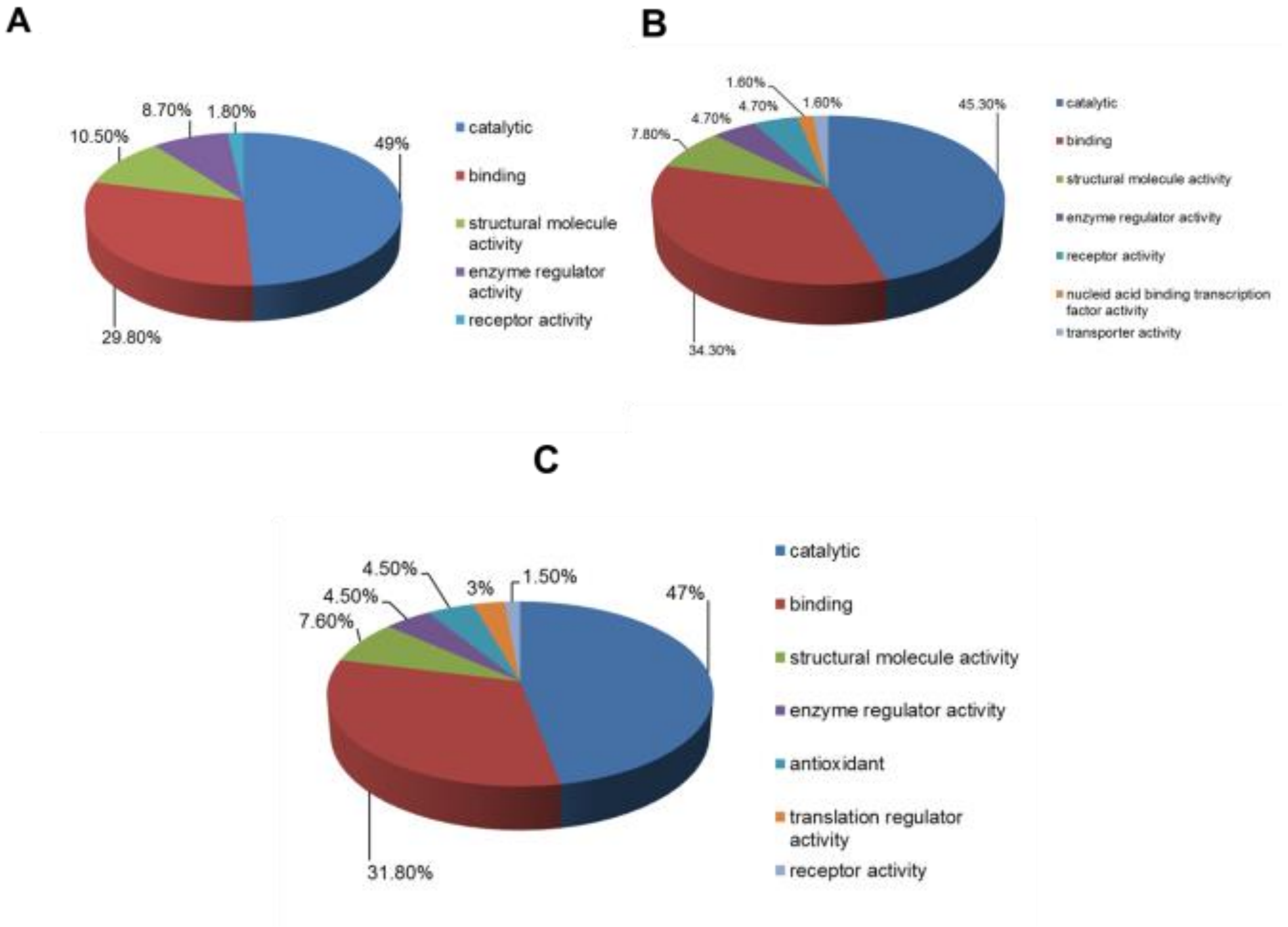
A
8.70%
1.80%
10.50%
49%
29.80%
catalytic
binding
structural molecule activity
enzyme regulator activity
receptor activity
B
1.60%
4.70%
1.60%
4.70%
7.80%
45.30%
34.30%
catalytic
binding
structural molecule activity
enzyme regulator activity
receptor activity
nucleid acid binding transcription factor activity
transporter activity
C
4.50%
3%
1.50%
4.50%
7.60%
47%
31.80%
catalytic
binding
structural molecule activity
enzyme regulator activity
antioxidant
translation regulator activity
receptor activity

S3 Fig.

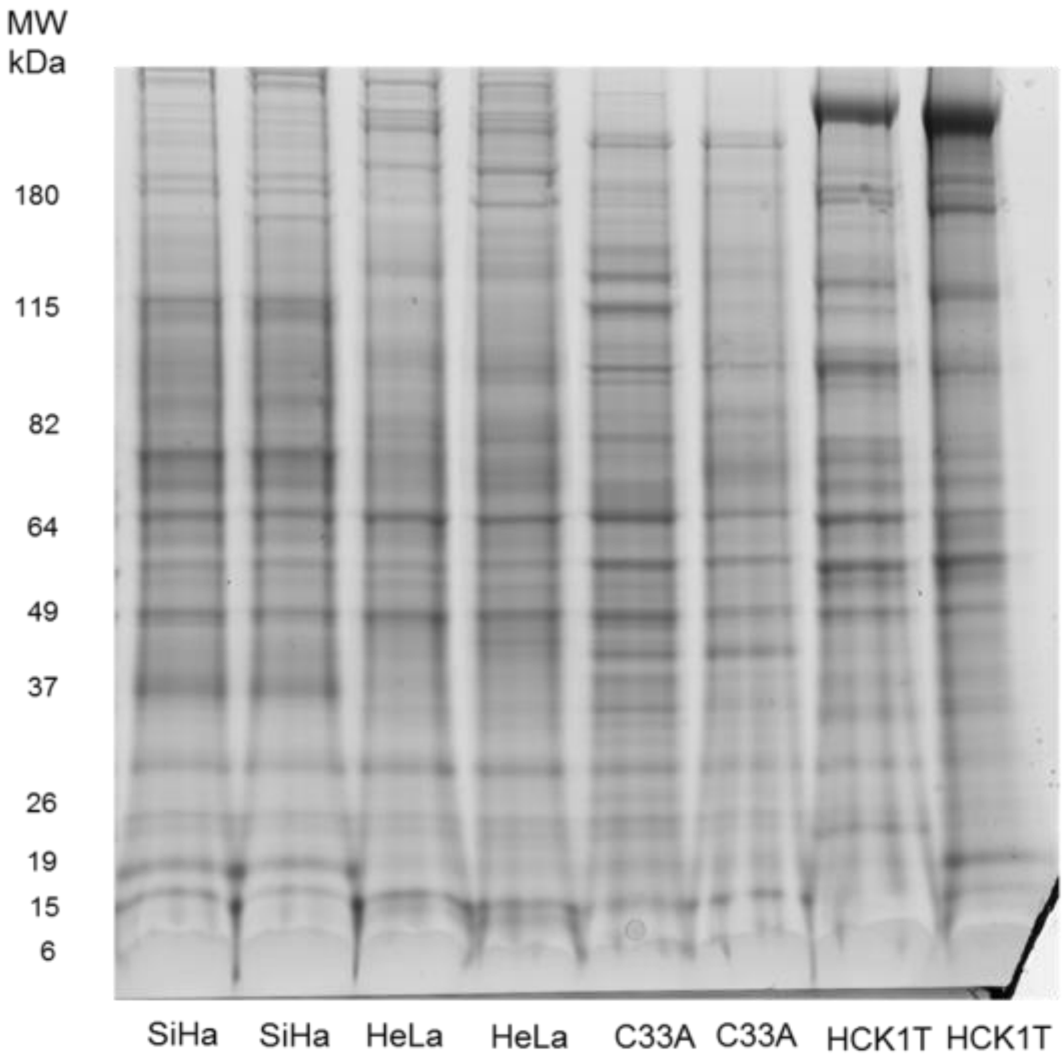
MW
kDa
180
115
82
64
49
37
26
19
15
6
SiHa
SiHa
HeLa
HeLa
C33A
C33A
HCK1T
HCK1T

S4 Fig.

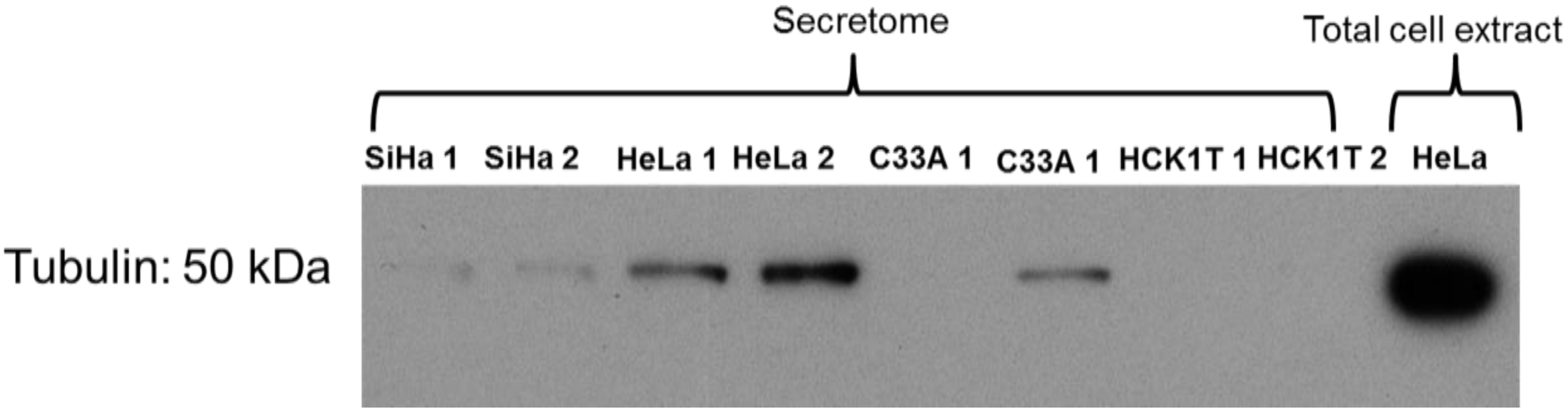
Secretome
Total cell extract
SiHa 1
SiHa 2
HeLa 1
HeLa 2
C33A 1
C33A 1
HCK1T 1
HCK1T 2
HeLa
Tubulin: 50 kDa

S5 Fig.

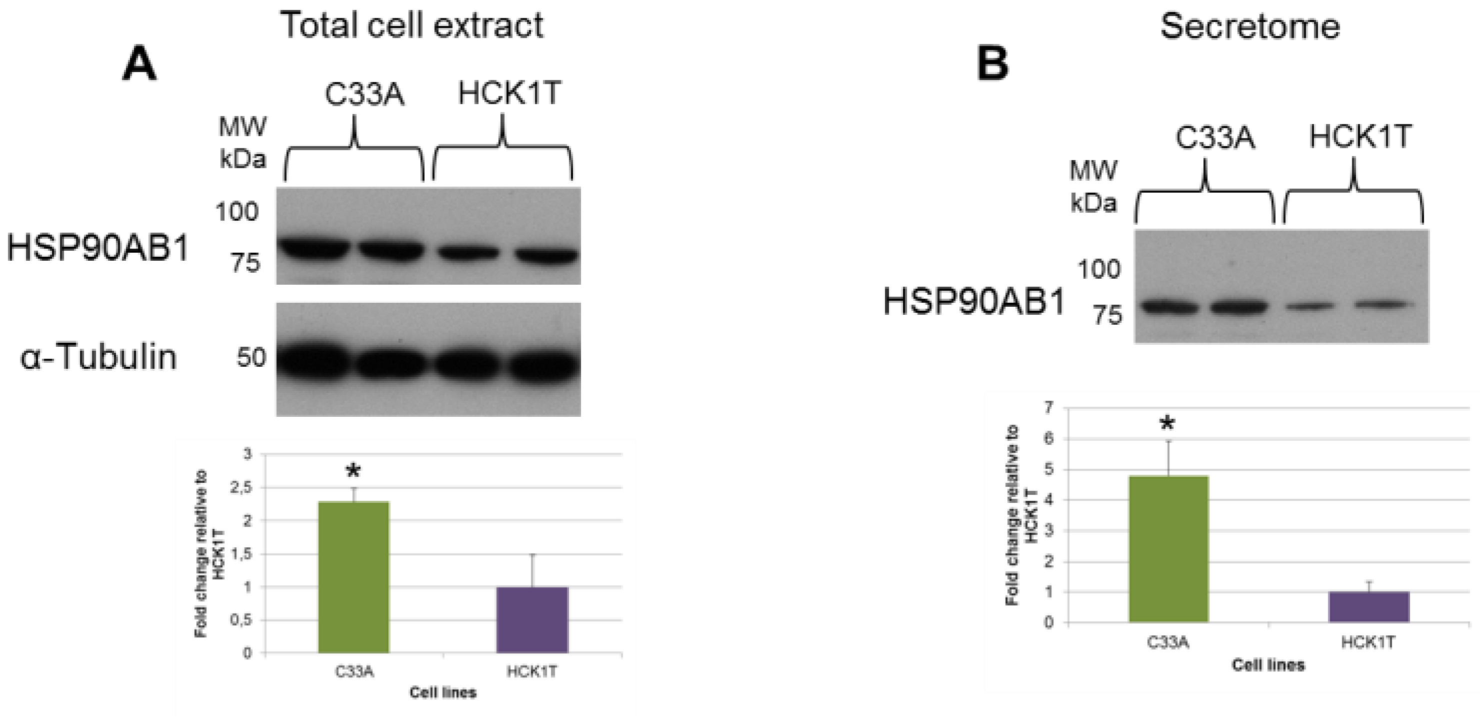

A
Total cell extract
C33A
HCK1T
MW kDa
HSP90AB1
100
75
α-Tubulin
50
Fold change relative to HCK1T
Cell lines
B
Secretome
C33A
HCK1T
MW kDa
HSP90AB1
100
75
Fold change relative to HCK1T
Cell lines
